# Effect on Treatment Adherence of Distributing Essential Medicines at No Charge: The CLEAN Meds Randomized Clinical Trial

**DOI:** 10.1001/jamainternmed.2019.4472

**Published:** 2019-10-07

**Authors:** Navindra Persaud, Michael Bedard, Andrew S. Boozary, Richard H. Glazier, Tara Gomes, Stephen W. Hwang, Peter Jüni, Michael R. Law, Muhammad M. Mamdani, Braden J. Manns, Danielle Martin, Steven G. Morgan, Paul I. Oh, Andrew D. Pinto, Baiju R. Shah, Frank Sullivan, Norman Umali, Kevin E. Thorpe, Karen Tu, Andreas Laupacis

**Affiliations:** 1 Department of Family and Community Medicine, University of Toronto, Toronto, Ontario, Canada; 2 Li Ka Shing Knowledge Institute, St Michael’s Hospital, Toronto, Ontario, Canada; 3 Department of Family and Community Medicine, St Michael’s Hospital, Toronto, Ontario, Canada; 4 Institute of Health Policy, Management, and Evaluation, University of Toronto, Toronto, Ontario, Canada; 5 Department of Family Medicine, Northern Ontario School of Medicine, Sudbury, Ontario, Canada; 6 Department of Health Policy and Management, Harvard School of Public Health, Boston, Massachusetts; 7 Dalla Lana School of Public Health, University of Toronto, Toronto, Ontario, Canada; 8 Institute for Clinical Evaluative Sciences, Toronto, Ontario, Canada; 9 Leslie Dan Faculty of Pharmacy, University of Toronto, Toronto, Ontario, Canada; 10 Applied Health Research Centre, St Michael’s Hospital, Toronto, Ontario, Canada; 11 Department of Medicine, University of Toronto, Toronto, Ontario, Canada; 12 Centre for Health Services and Policy Research, School of Population and Public Health, The University of British Columbia, Vancouver, British Columbia, Canada; 13 Centre for Healthcare Analytics Research and Training, St Michael’s Hospital, Toronto, Ontario, Canada; 14 Department of Community Health Sciences, Cumming School of Medicine, University of Calgary, Calgary, Alberta, Canada; 15 Department of Medicine, Cumming School of Medicine, University of Calgary, Calgary, Alberta, Canada; 16 O’Brien Institute for Public Health, Cumming School of Medicine, University of Calgary, Calgary, Alberta, Canada; 17 Libin Cardiovascular Institute, Cumming School of Medicine, University of Calgary, Calgary, Alberta, Canada; 18 Women’s College Hospital Institute for Health Systems Solutions and Virtual Care, Women’s College Hospital, Toronto, Ontario, Canada; 19 School of Population and Public Health, University of British Columbia, Vancouver, British Columbia, Canada; 20 Toronto Rehabilitation Institute, University Health Network, Toronto, Ontario, Canada; 21 The Upstream Lab, MAP Centre for Urban Health Solutions, St Michael’s Hospital, Toronto, Ontario, Canada; 22 Department of Research and Innovation, North York General Hospital, Toronto, Ontario, Canada; 23 Division of Population and Behavioral Science, University of St Andrews, St Andrews, Scotland

## Abstract

**Importance:**

Nonadherence to treatment with medicines is common globally, even for life-saving treatments. Cost is one important barrier to access, and only some jurisdictions provide medicines at no charge to patients.

**Objective:**

To determine whether providing essential medicines at no charge to outpatients who reported not being able to afford medicines improves adherence.

**Design, Setting, and Participants:**

A multicenter, unblinded, parallel, 2-group, superiority, outcomes assessor–blinded, individually randomized clinical trial conducted at 9 primary care sites in Ontario, Canada, enrolled 786 patients between June 1, 2016, and April 28, 2017, who reported cost-related nonadherence. Follow-up occurred at 12 months. The primary analysis was performed using an intention-to-treat principle.

**Interventions:**

Patients were randomly allocated to receive free medicines on a list of essential medicines in addition to otherwise usual care (n = 395) or usual medicine access and usual care (n = 391).

**Main Outcomes and Measures:**

The primary outcome was adherence to treatment with all medicines that were appropriately prescribed for 1 year. Secondary outcomes were hemoglobin A_1c_ level, blood pressure, and low-density lipoprotein cholesterol levels 1 year after randomization in participants taking corresponding medicines.

**Results:**

Among the 786 participants analyzed (439 women and 347 men; mean [SD] age, 51.7 [14.3] years), 764 completed the trial. Adherence to treatment with all medicines was higher in those randomized to receive free distribution (151 of 395 [38.2%]) compared with usual access (104 of 391 [26.6%]; difference, 11.6%; 95% CI, 4.9%-18.4%). Control of type 1 and 2 diabetes was not significantly improved by free distribution (hemoglobin A_1c_, −0.38%; 95% CI, −0.76% to 0.00%), systolic blood pressure was reduced (−7.2 mm Hg; 95% CI, −11.7 to −2.8 mm Hg), and low-density lipoprotein cholesterol levels were not affected (−2.3 mg/dL; 95% CI, −14.7 to 10.0 mg/dL).

**Conclusions and Relevance:**

The distribution of essential medicines at no charge for 1 year increased adherence to treatment with medicines and improved some, but not other, disease-specific surrogate health outcomes. These findings could help inform changes to medicine access policies such as publicly funding essential medicines.

**Trial Registration:**

ClinicalTrials.gov identifier: NCT02744963

## Key Points

**Question:**

Does providing a comprehensive set of essential medicines at no charge to primary care patients who have difficulty affording medicines improve treatment adherence?

**Findings:**

In this randomized clinical trial of 786 primary care patients, free distribution of essential medicines vs usual access resulted in greater adherence to treatment with medicines (absolute risk difference, 11.6%). Control of type 1 and 2 diabetes was not significantly improved by free distribution of essential medicines (hemoglobin A_1c_, −0.38%), systolic blood pressure was reduced (−7.2 mm Hg), and low-density lipoprotein cholesterol levels were not affected (−2.3 mg/dL).

**Meaning:**

Distributing essential medicines at no charge increased adherence to appropriately prescribed treatment with medicines and improved some disease-specific surrogate health outcomes.

## Teaser

This randomized clinical trial examines whether providing essential medicines at no charge to outpatients who reported not being able to afford medicines improves treatment adherence.

## Introduction

Lifesaving medicines such as treatments for cardiovascular disease and HIV and AIDS are often not accessible because of the cost to patients, among other reasons.^1^ Global estimates of medication nonadherence are between 40% and 60% for treatments for chronic diseases.^2^ A systematic review of interventions to improve adherence identified few effective interventions and underscored the need for additional high-quality studies.^3^ Providing certain medicines at no charge for people with specific conditions such as myocardial infarction, hypertension, HIV and AIDS, and schizophrenia can improve surrogate and direct health measures related to those conditions.^4,5,6,7^ The World Health Organization recommends that countries develop a list of essential medicines “that satisfy the priority health care needs of the population” for the purpose of increasing access and quality of care, and facilitating medicine access is one way to help achieve universal health coverage.^8^

Previous studies such as the RAND Health Insurance Experiment did not isolate the effect of medicine access from that of health care access.^9^ Canada is a suitable setting to measure the effects of medicine distribution because physician care and hospitalizations are universally publicly funded, while medicines are not.^10^ Public coverage of medicines used outside of Canadian hospitals varies by province, but is most frequently offered for specific groups, such as people receiving social assistance and those older than 65 years.^10^ Approximately 55% of people in Ontario, Canada, have employer-based private insurance plans that typically cover medicines with copayments or deductibles, while approximately 28% have public insurance that also involve copayments and deductibles.^11^

We conducted a randomized clinical trial examining the effect of providing Canadian outpatients who reported not being able to afford medicines with free distribution of essential medicines. Because the provided medicines included only treatments that have been well established as effective—such as those for cardiovascular disease, HIV and AIDS, and pneumonia—adherence was chosen as the primary outcome, and surrogate health outcomes were the secondary outcomes.

## Methods

### Study Design

The Carefully Selected and Easily Accessible at No Charge Medicines (CLEAN Meds) trial is a multicenter, unblinded, parallel, 2-group, superiority, outcomes assessor–blinded, individually randomized clinical trial with 1:1 allocation conducted at 9 primary care sites in Ontario, Canada, that enrolled patients between June 1, 2016, and April 28, 2017. The trial protocol is available in Supplement 1. The trial was registered with ClinicalTrials.gov (NCT02744963) and a protocol has been published.^12^ The trial is reported in accordance with the 2010 CONSORT statement^13^ and the intervention is described using the TIDieR (Template for Intervention Description and Replication) checklist.^14^ After registration with ClinicalTrials.gov, publication of the protocol, and initiation of the study, the duration of the trial was extended from 12 to 24 months when additional funding was obtained (trial protocol in Supplement 1). For the analysis of the primary outcome, we originally planned to use electronic pill bottle cap devices in one-seventh of participants to confirm adherence measurements, but owing to a large amount of missing data in both groups we removed this measure from the definition of the primary outcome; we report the results when the available electronic pill bottle cap device data were used in eTable 1 in Supplement 2. A data and safety monitoring board met to ensure that medication incidents were properly addressed and that the intervention was not harming participants. Ethics approval for the conduct of this study was obtained from the St Michael’s Research Ethics Board, the Huron Shores Family Health Team Research Ethics Committee, and the Laurentian University Research Ethics Board. All enrolled participants provided written informed consent.

### Patients

Patients 18 years or older who self-reported medication nonadherence owing to cost in the last 12 months were eligible for inclusion. After potentially eligible participants were identified by clinicians at routine visits, study personnel asked a question adapted from the Commonwealth Fund International Health Policy Survey to confirm cost-related nonadherence: “In the last twelve months, did you not fill a prescription or do anything to make a prescription last longer because of the cost?”^15^^(p30)^ We excluded family members living at the same address as participants already enrolled in the study to avoid contamination and excluded patients who joined the clinic within the last 6 months to deter patients from joining the practice to enroll in the study.

### Trial Procedures

Patients who met the eligibility criteria were randomly allocated to 1 of 2 groups. The intervention group received free distribution of essential medicines. The control group accessed medications as usual. Randomization and allocation concealment were achieved through a web-based tool that was accessed through the REDCap electronic case report forms application and was stratified by center and blocked using permuted blocks of 2 to 4.^16^

Patients in the intervention group received free distribution of medicines on a list of essential medicines as well as otherwise usual care. The list of 128 essential medicines was adapted from the 2013 World Health Organization Model List of Essential Medicines based on Canadian clinical practice guidelines,^17^ suggestions from clinicians and patients, prescribing volumes, and evidence syntheses (eAppendix 1 in Supplement 2).^18^ The medicines included treatments for acute conditions (eg, antibiotics and analgesics) and chronic conditions (eg, antipsychotics, antiretrovirals, glucose-lowering medicines, and antihypertensives). Patients could be prescribed medicines that were not on the essential list and could access them in the usual way (eg, by paying for them). Participants could be switched from a medicine not on the list to an equivalent that was on the list. Medicine distribution was primarily through the mail to an address of the participant’s choice. A community pharmacist (N.U.; with a Bachelor of Science in Pharmacy and more than 15 years of experience) contacted patients by telephone from a pharmacy established for the study. Medicines that needed to be started in a timely fashion (eg, anti-infectives, analgesics, diuretics, bronchodilators, antihypertensives, and antipsychotics) were also stored at each study site for dispensing by clinicians. Controlled substances (eg, opioids, sedatives, and stimulants) were not included in the intervention; patients accessed these medications in the usual fashion.

Participants allocated to the control group had their usual access to medicines. Typical annual out-of-pocket costs to participants in the control group were the full cost of medicines for those with no insurance (eg, approximately $800, as well as dispensing fees of approximately $10 per prescription for a patient taking oral diabetes medicines), a deductible for an older adult with public drug coverage (eg, $100 plus copayments of $4 for each prescription filled), or a percentage of total medicine costs for those with private insurance (eg, $160 = $800 × 20%, assuming 80% coverage). Medicines were generally dispensed by community pharmacies, some of which offer local delivery services for medicines on request.

### Outcomes

The follow-up period was 12 months. The prespecified primary outcome was adherence to all appropriately prescribed medicines.^19^ The primary outcome was determined at 12 months by assessing whether each prescription was both appropriate (based on explicit criteria) and taken as prescribed for at least 80% of expected doses.^19^ A participant was either classified as receiving only appropriate prescriptions and being adherent to all of them (evidence of taking at least 80% of expected doses), or as having either received at least 1 potentially inappropriate prescription or being nonadherent to at least 1 medicine. We reviewed primary care prescribing records to determine whether each prescription was potentially inappropriate using established criteria based only on the prescribed medicines (eAppendix 2 in Supplement 2).^20^ We considered as appropriate all prescriptions that did not meet criteria for being potentially inappropriate. Two adjudicators independently applied the explicit criteria in a blinded fashion; there were no disagreements. For adherence, we used the lowest estimate from the 2 methods used: reviews of primary care records for prescription renewal intervals during the 12-month study period and patient report of the number of doses missed during the last week, as reported by telephone interview or email survey between 9 and 12 months. Blinded abstraction of medical records was done by 1 adjudicator from a team of 5 and verified by another after a training period for each adjudicator.

The prespecified secondary outcomes were the proportion of medicines that were appropriately prescribed, the proportion of medicines that met adherence criteria, hemoglobin A_1c_ levels in patients treated for type 1 or 2 diabetes (adjusted for baseline), blood pressure in patients treated with an antihypertensive (adjusted for baseline), and low-density lipoprotein cholesterol levels in patients treated with a statin (adjusted for baseline). Hemoglobin A_1c_, blood pressure, and low-density lipoprotein cholesterol levels were obtained by review of medical records during the baseline period (up to 3 months prior to randomization) and at follow-up (9-12 months after randomization). Clinicians ordered investigations as usual in both groups; no instructions related to clinical care were provided by the trial team. To compare experiences between groups, after a 9- to 12-month follow-up period we asked patients 14 questions about their care, medicine dispensing, and social circumstances (eTable 2 in Supplement 2). Serious adverse events, including hospitalizations and deaths, were ascertained through clinician reports, patient reports, and reviews of the primary care medical records.

### Statistical Analysis

The sample size was calculated to detect a 10% absolute difference (80% power, type I error of 5%) in the primary outcome of appropriate adherence and assuming that 40% to 60% of patients in the control group would be appropriately adherent to all medications.^4,6,21^ A sample size of 392 per group was required. The primary analysis was performed using an intention-to-treat principle. Appropriate adherence was compared using a χ^2^ test and the unadjusted treatment effect was expressed as the absolute risk difference. For the primary analysis, *P* < .05 was used to reject the null hypothesis of no difference. We did not correct for multiple comparisons because comparisons other than for the prespecified primary and secondary outcomes are hypothesis generating. For the exploratory subgroup analysis, we fit a logistic regression model to determine whether the following characteristics modified the effect of the intervention: age, sex, urban vs rural site, number of baseline medicines, and income.

## Results

### Patients

Between June 1, 2016, and April 28, 2017, 1130 individuals identified as potentially eligible by clinicians were assessed for eligibility and 786 were randomly allocated (Figure). The characteristics of participants in the 2 groups are summarized in Table 1.^22^ For the 22 of 786 participants (2.8%) who withdrew consent—9 of 395 (2.3%) in the free distribution group and 13 of 391 (3.3%) in the usual access group—data collected prior to withdrawal were included in the analysis.

**Figure.**
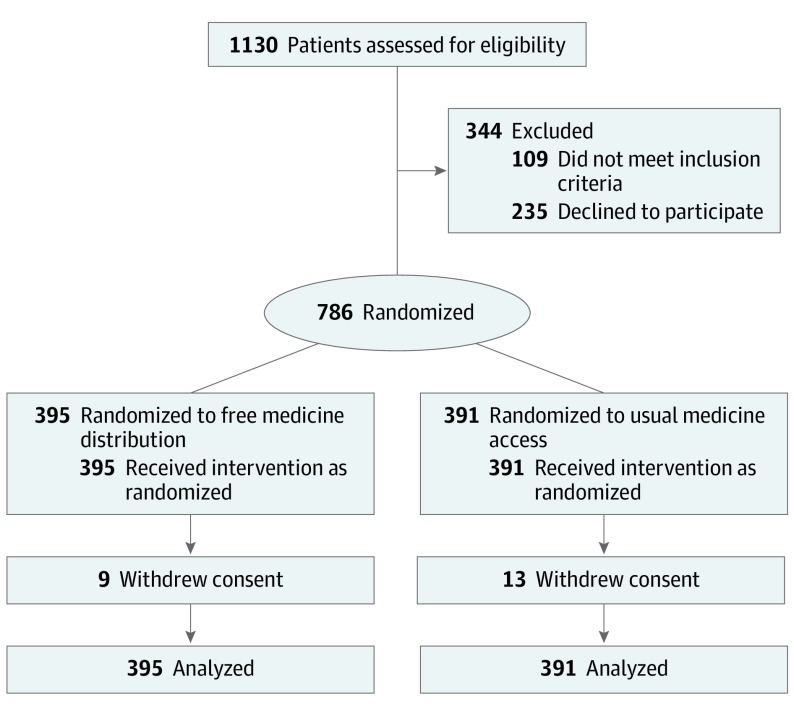
Participant Flow Diagram

**Table 1.** Baseline Participant Characteristics by Group

Characteristic	Participants, No. (%)	
	Free Distribution Group (n = 395)	Usual Access Group (n = 391)
Female sex	220 (55.7)	219 (56.0)
Age, mean (SD), y	51.0 (14.2)	50.4 (14.3)
Age ≥65 y	71 (18.0)	64 (16.4)
Race/ethnicity		
White	256 (64.8)	260 (66.5)
Black	35 (8.9)	39 (10.0)
Southeast or East Asian (including Korean, Japanese, Filipino, and Chinese)	28 (7.1)	19 (4.9)
South Asian	25 (6.3)	24 (6.1)
Latin American	10 (2.5)	15 (3.8)
Indigenous	12 (3.0)	14 (3.6)
West Asian (including Arab)	6 (1.5)	5 (1.3)
Mixed or other	22 (5.6)	8 (2.0)
Declined to provide	1 (0.3)	7 (1.8)
Main income source		
Wages and salaries (including self-employed)	218 (55.2)	221 (56.5)
Pension	50 (12.7)	42 (10.7)
Social support (eg, welfare or disability)	36 (9.1)	47 (12.0)
Unemployment insurance	15 (3.8)	9 (2.3)
Other	56 (14.2)	51 (13.0)
Declined to provide	20 (5.1)	21 (5.4)
Household income, Can$^a^		
<30 000	205 (51.9)	182 (46.5)
30 000-70 000	92 (23.3)	99 (25.3)
>70 000	21 (5.3)	22 (5.6)
Declined to provide	77 (19.5)	88 (22.5)
No. of medicines prescribed at baseline, mean (SD)	5.3 (3.6)	5.6 (4.0)
Site		
Urban	269 (68.1)	267 (68.3)
Rural	126 (31.9)	124 (31.7)
Prescribed		
Diabetes treatment	89 (22.5)	91 (23.3)
Antihypertensive	122 (30.9)	114 (29.2)
Statin	81 (20.5)	81 (20.7)

^a^ The median Canadian household income in 2015 was $70 336, and the poverty line was $37 542.^22^

The categories of medicines prescribed were similar between groups (Table 2). Commonly prescribed medicines during the study period included analgesics, diabetes treatments, proton pump inhibitors, treatments for hypertension or vascular disease, and treatments for asthma or chronic obstructive pulmonary disease.

**Table 2.** Participants Prescribed Medicines by Anatomical Therapeutic Chemical Classification System Main Groups

Anatomical Therapeutic Chemical Main Group (Examples of Medicines Commonly Prescribed)	Prescriptions, No. (%)	
	Free Distribution Group (n = 2071)	Usual Access Group (n = 2183)
Nervous system (gabapentin, sertraline, venlafaxine, and acetaminophen)	424 (20.5)	450 (20.6)
Alimentary tract and metabolism (metformin, pantoprazole, rabeprazole, and insulin)	381 (18.4)	403 (18.5)
Cardiovascular system (atorvastatin, ramipril, rosuvastatin, amlodipine, and hydrochlorothiazide)	326 (15.7)	366 (16.8)
Respiratory system (albuterol, fluticasone, and tiotropium)	274 (13.2)	264 (12.1)
Dermatologic (hydrocortisone and betamethasone)	161 (7.8)	159 (7.3)
Blood and blood-forming organs (acetylsalicylic acid and ferrous fumarate)	125 (6.0)	140 (6.4)
Musculoskeletal system (naproxen and ibuprofen)	117 (5.6)	128 (5.9)
Genitourinary system and sex hormones (estradiol)	116 (5.6)	124 (5.7)
Anti-infectives for systemic use (amoxicillin)	86 (4.2)	88 (4.0)
Systemic hormonal preparations (levothyroxine)	40 (1.9)	37 (1.7)
Other	21 (1.0)	24 (1.1)

### Adherence

Free distribution increased the number of participants in the free distribution group who were appropriately adherent to all medicines, compared with those in the usual access group (151 of 395 [38.2%] vs 104 of 391 [26.6%]; difference, 11.6%; 95% CI, 4.9%-18.4%; *P* < .001) (Table 3). We performed sensitivity analyses using less stringent definitions and found that the difference was similar in magnitude and remained statistically significant (eTable 1 in Supplement 2). There was little indication of subgroup effects: no effect modification terms were statistically significantly different (age, sex, urban vs rural site, number of baseline medicines, and income); the overall test of including effect modification was not statistically significant; and the estimate of the effect of the intervention on adherence was similar without adjustment for baseline characteristics (odds ratio, 1.7; 95% CI, 1.3-2.3) and with adjustment (odds ratio, 1.7; 95% CI, 1.2-2.3).

**Table 3.** Primary and Secondary Outcome Results by Group

Outcome	Free Distribution Group (n = 395)	Usual Access Group (n = 391)	Difference, % (95% CI)	*P* Value
Primary outcome, No. (%)				
Participants appropriately adherent to all medicines	151 (38.2)	104 (26.6)	11.6 (4.9 to 18.4)	<.001
Secondary outcomes, %				
Mean % of medicines adhered to by each participant^a^	66.1	56.4	7.2 (1.1 to 14.0)	.02
Mean % of medicines potentially inappropriately prescribed to each participant^a^	0.17	0.85	−0.66 (−0.79 to −0.33)	.007

^a^ Differences estimated from rate ratios and estimated mean percentage in control group. Rate ratio for medicines adhered to was 1.13 (95% CI, 1.02-1.25). Rate ratio for potentially inappropriate prescriptions was 0.22 (95% CI, 0.064-0.60).

### Secondary Outcomes

The proportion of medicines each participant was adherent to was higher in those receiving free distribution than those with usual access (66.1% vs 56.4%; difference, 7.2%; 95% CI, 1.1%-14.0%; *P* = .02) and the proportion of potentially inappropriately prescribed medicines was lower in those with free distribution than those with usual access (0.17% vs 0.85%; difference, −0.66%; 95% CI, −0.79% to −0.33%; *P* = .007) (Table 3). Free distribution improved systolic blood pressure among those prescribed an antihypertensive drug compared with those receiving usual access (−7.2 mm Hg; 95% CI, −11.7 to −2.8 mm Hg; *P* = .002), but did not have a statistically significant effect on the other surrogate health outcomes such as low-density lipoprotein cholesterol level (−2.3 mg/dL; 95% CI, −14.7 to 10.0 mg/dL; *P* = .70 [to convert to millimoles per liter, multiply by 0.0259]), although there was a nonsignificant decrease in hemoglobin A_1c_ levels in participants in the free distribution group who were prescribed a diabetes treatment (−0.38%; 95% CI, −0.76% to 0.00%; *P* = .05 [to convert to proportion of total hemoglobin, multiply by 0.01]) (Table 4).

**Table 4.** Secondary Surrogate Health Outcome Results by Group

Outcome	Free Distribution Group	Usual Access Group
Hemoglobin A_1c_, %		
No.	73	68
Baseline, mean (SD)	8.20 (1.86)	8.15 (1.85)
Follow-up, mean (SD)	7.69 (1.50)	8.04 (1.58)
Difference (95% CI)	−0.38 (−0.76 to 0.00)	NA
*P* value	.05	NA
Systolic blood pressure, mm Hg		
No.	105	88
Baseline, mean (SD)	137 (19)	135 (17)
Follow-up, mean (SD)	132 (16)	139 (19)
Difference (95% CI)	−7.2 (−11.7 to −2.8)	NA
*P* value	.002	NA
Diastolic blood pressure, mm Hg		
No.	105	88
Baseline, mean (SD)	81 (13)	81 (11)
Follow-up, mean (SD)	78 (12)	80 (13)
Difference (95% CI)	−2.0 (−5.0 to 1.0)	NA
*P* value	.19	NA
LDL cholesterol level, mg/dL		
No.	48	40
Baseline, mean (SD)	88.9 (38.7)	77.3 (34.8)
Follow-up, mean (SD)	81.2 (34.8)	81.2 (42.5)
Difference (95% CI)	−2.3 (−14.7 to 10.0)	NA
*P* value	.70	NA

Abbreviations: LDL, low-density lipoprotein; NA, not applicable.

SI conversion factors: To convert LDL cholesterol to millimoles per liter, multiply by 0.0259; hemoglobin A_1c_ to proportion of total hemoglobin, multiply by 0.01.

There were statistically significant differences between groups in 10 of the 14 patient-oriented outcomes (eTable 2 in Supplement 2). Compared with the time of enrollment, participants in the free distribution group were more likely than those in the usual care group to report receiving their medicines before the previous prescription ran out (217 of 261 [83.1%] vs 157 of 237 [66.2%]; difference, 16.9%; 95% CI, 9.0%-24.8%), more likely to report that their care improved (123 of 266 [46.2%] vs 47 of 251 [18.7%]; difference, 27.5%; 95% CI, 19.4%-35.6%), and more likely to report being able to “make ends meet” or afford necessities (230 of 266 [86.5%] vs 79 of 238 [33.2%]; difference 53.3%; 95% CI, 45.6%-60.9%). There was no statistically significant difference between groups in the following 5 of 14 patient oriented outcomes: receiving medicines in good condition, receiving new medicines quickly, reported medicine adverse effects, having unanswered questions about medicines, information changing the way medicines were taken, and the information from the pharmacist and prescriber matching.

### Safety

There was no substantial difference between participants in the free distribution group and those in the usual access group in serious adverse events (33 of 395 [8.4%] vs 35 of 391 [9.0%]; *P* = .80). Hospitalizations (26 of 395 [6.6%] vs 25 of 391 [6.4%]; *P* > .99), deaths (8 of 395 [2.0%] vs 8 of 391 [2.0%]; *P* > .99), and other serious adverse events (7 of 395 [1.8%] vs 4 of 391 [1.0%]; *P* = .55) were also similar between the free distribution group and the usual access group. There were 37 medication incidents involving 32 of 395 participants (8.1%) receiving free distribution.

## Discussion

In our multicenter randomized trial, distributing a comprehensive set of essential medicines at no charge improved adherence. Free distribution also lowered systolic blood pressure and there was a suggestion of better diabetes control, although results did not reach statistical significance. There was no effect on low-density lipoprotein cholesterol levels. There was no increase in potentially inappropriate prescribing and there was no substantial difference in serious adverse events. Participants receiving free medicine distribution were more likely to report being able to make ends meet; the hypothesis that medicine access allows people to afford other necessities can be tested in future studies.

To our knowledge, no previous trial has assessed the effect of providing a wide range of medicines for free, including treatments for chronic noncommunicable disease (eg, diabetes and rheumatoid arthritis), chronic infectious disease (eg, HIV and AIDS), and acute conditions (eg, pneumonia) and symptoms (eg, pain). A trial of free access to secondary prevention medicines after a myocardial infarction found greater adherence in a higher risk population (38.9% in usual access patients, compared with 26.6% in our study) and modest improvements with free access (absolute increase, 5.4%).^4^ Offsetting copayments for clopidogrel bisulfate or ticagrelor after an acute myocardial infarction increased adherence slightly (absolute increase, 3.3%) but did not affect major adverse cardiovascular events.^23^ Although improving medicine adherence is difficult—only 5 of 17 complex interventions involving frequent patient contact modestly improved adherence^3^—even relatively small increases in adherence to effective treatments seem to improve health outcomes such as cardiovascular events or reduce mortality such as HIV- and AIDS-related mortality in higher risk individuals.^4,7^

Health improvements with free distribution are supported by the changes in some surrogate health outcomes. The mean systolic blood pressure reduction of 7.2 mm Hg observed here is likely large enough to reduce mortality at some baseline levels.^24^ The observed small reduction in hemoglobin A_1c_ of 0.38%, if true, may be enough to reduce microvascular complications, based on trials of intensive control.^25^ At the same time, many patients did not see improvements in surrogate outcomes despite free distribution of medicines, emphasizing that cost is only one of several contributors to nonadherence and that medicines are just one part of care.

The setting and design of the trial allows inferences about the causal effects of essential medicine access to be drawn because all participants had access to publicly funded health care services. The study population included people with a range of income levels and sources and different ethnicities who lived in urban and rural settings.

### Limitations

Findings from one high-income country should be applied with caution in jurisdictions with different health care services and disease burdens. All the participants self-reported cost-related nonadherence, and the effect of free medicine distribution will presumably be smaller where adherence is better. At the same time, all participants had the option to apply for public insurance (which would have capped drug payments at approximately 4% of annual income) and others had public or private insurance, so the free distribution may be beneficial even in populations with some insurance. We did not measure adherence at baseline and so cannot determine if free distribution depends on the baseline level of adherence. Unblinded allocation to free medicine distribution could have motivated participants to exaggerate their adherence or resulted in different care from clinicians. The respective contributions of the different aspects of this multifaceted intervention, including free access and mailing of prescriptions, were not assessed in this 2-group trial, although mailing medicines is associated with improved adherence.^26^ There is no ideal method for determining medicine adherence because patient reports can overestimate adherence and objective measures such as medical record reviews can underestimate it. We could not confirm adherence with electronic pill bottle cap devices in a subset of participants as planned because most participants did not return the devices. There were missing data for the surrogate health outcomes as we did not provide clinicians with any instructions about checking blood pressure or laboratory indexes because doing so might have affected the outcomes. The cost-effectiveness of the intervention will be assessed after the 24-month trial is complete; a previous study has estimated that purchasing these medicines in bulk for the entire Canadian population would save approximately $4 billion per year, with an incremental government cost of approximately $1 billion per year.^27^ This study was not designed or powered to assess effects on mortality or morbidity, although the provided medicines include highly effective ones.

## Conclusions

In this randomized clinical trial, distributing essential medicines at no charge improved adherence to treatment with effective medicines. These results could help inform policy changes such as publicly funding a list of essential medicines as recommended by the Canadian Advisory Council on the Implementation of National Pharmacare.^28^

## Notes

Supplement 1. **Trial Protocol**

Supplement 2. **eAppendix 1.** Essential Medicines Provided to Patients With Free Distribution **eAppendix 2.** Criteria for Potentially Inappropriate Prescriptions **eTable 1.** Alternative Definitions of Primary Outcome Results Table **eTable 2.** Patient Oriented Outcome Results Table

Supplement 3. **Data Sharing Statement**
