# Supplementary material for: Effect on Treatment Adherence of Distributing Essential Medicines at No Charge: The CLEAN Meds Randomized Clinical Trial
Source: JAMA Intern Med. 2019 Oct 7;180(1):27–34. doi: 10.1001/jamainternmed.2019.4472 (PMC6784757; doi:10.1001/jamainternmed.2019.4472)
Supplement: Supplement 1. — Trial Protocol [file jamainternmed-180-27-s001.pdf]

CLEAN Meds RCT: Carefully seLected and Easily Accessible at No charge Medications

Canada is the only high income country where health care services are publicly funded for the general population but medications are not.[1] We propose an RCT of providing carefully selected, essential medications at no charge (compared with usual medication access) to primary care patients. The impact of this intervention on appropriate medication adherence, valid surrogate health outcomes and costs will be determined:

Population – Adult primary care patients who report cost-related non-adherence to medications
Intervention – Free and convenient access to a carefully selected set of essential medications with otherwise usual care (n = 392)
Control – Usual access to medications and usual care (n = 392)
Outcome – *Primary*: Appropriate adherence to medications over 12 months. Secondary: HbA1c levels (in patients with diabetes); blood pressure (in hypertensive patients); cholesterol (in patients with dyslipidemia); average per person medication costs.

1. The Need for a Trial

1.1 What is the problem to be addressed?

A large number of different medications are prescribed in Canada and a much larger number of Canadians do not take prescribed medications because of the cost. The intervention in this trial – the free provision of a carefully selected set of medications – has the potential to address both medication selection and medication adherence.

Medication adherence is an important determinant of health outcomes.[2] Approximately one half of treatments for chronic disease are not taken as directed.[3] Cost is one of several important causes of poor adherence and it is particularly important for vulnerable people.[4, 5] At least 2.4 million adult Canadians report cost related nonadherence to medications.[5] 31 % of prescriptions in Quebec are not filled and those of highest cost are least likely to be filled.[4] Providing full drug coverage may improve medication adherence and clinical outcomes, particularly for vulnerable individuals and those with chronic diseases.[6]

The size of many Canadian formularies such as the Ontario Drug Benefit Formulary, which contains over 3800 medications[7], makes it difficult for clinicians to know the safest and most effective medications to prescribe. This may partially explain poor medication selection. For example 16% of older Canadian primary care patients receive at least one potentially inappropriate medication.[8] 17% of NSAID prescriptions are for diclofenac[9] even though it is associated with additional vascular risk compared to other NSAIDs such as naproxen and has no advantage.[10] Inappropriate prescribing has been linked to excess morbidity and hospitalization.[8]

Approximately 12 billion dollars are spent each year by provincial and territorial governments on medications in Canada.[11] From 2000 to 2010, the annual average per capita growth in prescription expenditures in Canada was approximately 4%.[1] In contrast, comparable countries such as the United Kingdom only experienced growth approximating 1%.[1] The large number of medications in formularies may contribute to the high prices of medications in Canada. Medication costs are lower in countries such as Sweden with limited formularies (similar to the list of essential medications that will be employed in this study).[12] Per capita medication costs in 2011 for Canada and Sweden were $771 and $477,

respectively.[13] Multiple national reports over several decades have concluded that a national pharmacare system would save billions of dollars annually from a societal perspective[1, 14, 15] yet provincial decision makers have cited cost considerations in deciding against making large formularies containing thousands of medications[7] available to all residents.[1] The use of a carefully selected set of medications might be dramatically less expensive, yet still provide the general population with access to the majority of medications they need.

Access to medications is dependent on local pharmacies. In Canada, the current dispersion of dispensing over multiple pharmacies is associated with a greater risk of severe adverse drug reactions in the elderly.[16] This may be due to incomplete communication among prescribers and dispensing pharmacists.[16] The use of an integrated prescriber-pharmacist medication dispensing system has showed great promise. In this system, the clinician and pharmacist both have access to the electronic medical record where prescriptions are entered. The pharmacist dispenses medications by mail to the patient and provides counseling over the telephone. Observational studies have demonstrated that medication adherence and efficiency are greater when patients are mailed prescription medications.[17-21] Observational studies have also found an association between mailing prescription medications and fewer emergency room visits[22] and better management of dyslipidemia.[23]

An intervention that combines three critical elements – free access, a carefully selected set of medications, and centralized distribution and mailing – is a logical focus for a randomized controlled trial that would provide important information for policy makers. Other short evidence-based lists of medications have been developed, but trials have demonstrated that merely educating clinicians about medication lists does not change practice or the medications patients actually receive.[24] In this intervention, prescribing will be driven to the selected medications because patients will have access to these medications at no expense. It would be impractical and expensive to give patients access to thousands of different medications at no charge; a short list of medications couples naturally with centralized medication distribution for this trial and is easily scalable if the trial results are positive. The individual components of the intervention could also be trialed if the results of this trial are positive.

1.2 What are the principal research questions to be addressed?

Primary question: Does providing convenient access to a carefully selected set of essential medications for free to adult primary care patients increase appropriate medication adherence?

Secondary questions:

Does providing convenient access to a carefully selected set of essential medications for free to adult primary care patients :

1) Increase appropriate prescribing?
2) Increase treatment adherence (regardless of whether prescriptions are appropriate)?
3) Improve HbA1c levels in patients with type 2 diabetes on oral hypoglycemic medications?
4) Improve blood pressure control in hypertensive patients on antihypertensive medications?
5) Improve LDL cholesterol levels in patients taking a statin?
6) Reduce per capita medication costs?

1.3 Why is a trial needed now?

Over the past several decades, multiple reports have called for national Canada-wide pharmacare or a publicly funded medication system that incorporates a national formulary.[1, 14, 25, 26] Romanow's report concluded that a unified formulary is necessary for collective bargaining

efforts because some provinces and territories are too small to individually negotiate prices, are subject to intense lobbying efforts from the pharmaceutical industry, and are often pressured to make poor decisions based on decisions made by other provinces.[25] A national formulary has been argued to be necessary to improve access, reduce costs, improve practice and to allow the implementation of universal Canadian pharmacare. [1] Yet no such system exists in Canada.

Some health system leaders have recently expressed openness to policy change. In December of 2014 the Ontario Minister of Health wrote in a newspaper op-ed that he "put the issue of national pharmacare on the front burner at the meeting with [his] provincial, territorial, and federal colleagues" and that he "was encouraged by federal Health Minister Rona Ambrose's commitment after raising the need for a national pharmacare program."[27] The results of this trial may inform pharmacare policy discussions and they will be communicated directly to decision makers provincially and federally.

1.4 Give references to any relevant systematic review and discuss the need for your trial.

A recent Cochrane systematic review on interventions for enhancing medication adherence included one RCT that examined the impact of providing full coverage for medications after a myocardial infarction.[28] In the MI FREEE trial, the absolute adherence for all patients in the full coverage intervention group (43.9 ± 33.7%) was 5.4% (95% CI, 3.6–7.2%; P<0.001) higher compared to the usual coverage control group (38.9 ± 32.7%).[29] There was no significant improvement in the primary composite clinical outcome of the rate of first readmission for a major vascular event or coronary revascularization (17.6 intervention versus 18.8 control; HR, 0.93; 95% CI, 0.82 to 1.04; P=0.21).[29] However, secondary clinical outcomes of rates of total major vascular events or revascularization (21.5 intervention versus 23.3 control; HR, 0.89; 95% CI, 0.90 to 0.99; P=0.03) and rates of first major vascular event (11.0 intervention versus 12.8 control; HR, 0.86; 95% CI, 0.74 to 0.99; P=0.03) were significantly reduced in the full coverage group.[29] The table below explains how our trial differs from the MI FREEE trial:

	**MI FREEE**	**Our trial**
Setting	United States, hospitalized patients	Canada, outpatient
Patients	Patients hospitalized for MI	Primary care patients who report cost-related non-adherence
Intervention	Coverage of medications that would ordinarily be prescribed	Provision of only a carefully selected set of medications for free
Medication classes	Cardiovascular only	Almost all
Randomization methodology	Cluster, plan level	Individual patient
Medication adherence methodology	Medication possession ratio	Patient self-report, electronic monitoring, prescription timing
Clinical outcomes	Vascular events and other cardiovascular outcomes	HbA1c levels, blood pressure, LDL

Our review of the literature and registries identified no other RCTs assessing the impact on adherence of providing medications at no charge. The table below summarizes the most relevant studies where the intervention or exposure included access to medications for free. In all studies, the intervention differs from the control condition in ways other than free access to medications, and participants in the control groups had access to medications without charge.

	**Thom (2013)**	**Farooq (2011)**	**Doshi (2009)**
Design	RCT	RCT	Cohort
Setting	England, Ireland, Netherlands and India	Pakistan	USA

Population	Adults at high risk for cardiovascular disease	Adults with schizophrenia and schizoaffective disorders	Adults with an indication for lipid lowering treatment
Intervention or Exposure	Fixed dose combination pill provided for free.<br>N=1,002	Medication adherence counselling for participants and primary care giver with treatment administration supervised directly by care giver and medications free of charge.<br>N= 55	Usual care with medications free of charge.<br>N= 495
Control	Usual care with the *option* to receive usual medications for free.<br>N= 1,002	Usual care with the *option* to receive usual medications for free.<br>N= 55	Increased medication copayments but medications *not* free of charge.<br>N= 5,109
Outcome	Adherence to treatment (defined as the number of days medications were taken in the week prior to visit via self-reporting during visits) and changes in SBP and LDL-C from baseline at 12 months.	Adherence to the treatment (complete adherence defined as participants always taking medication as prescribed without any break during the assessment period via patient interview and pill counts) and changes in symptoms (Positive and Negative Syndrome Scale for Schizophrenia -PANSS) and functional (Global Assessment of Functioning - GAF) scores from baseline at 12 months.	Adherence to treatment defined as the proportion of days covered (the number of days with lipid-lowering drug supply on hand divided by the number of days in the specified time period) with lipid-lowering medications >=80% at 24 months.
Results	• Intervention group had better adherence and were more likely to adhere vs control group (829 (86%) vs 621 (65%); Relative Risk of being adherent, 1.33; 95% CI,1.26, 1.41; P<0.001)<br>• Interventional group had better clinical outcomes than the control group; reductions in SBP (−2.6mmHg; 95%CI, −4.0 to −1.1mmHg; P<0.001) and LDL-C (−4.2mg/dL; 95%CI, −6.6, −1.9mg/dL; P<0.001)	• Intervention group had better adherence and were more likely to adhere than control group (complete adherence: 37 (67.3%) vs 25 (45.5%); Relative Risk of being adherent 1.59; 95% CI, 1.03, 2.53; P<0.02).<br>• Interventional group had significant improvement in symptoms (PANSS Total scores: time effect P=0.017, between participant effect P=0.003 and PANSS Positive score: time effect P=0.011, between participants effect; P=0.003)	• Control group had significantly decreased adherence compared to the exposure group (-19.2% and -19.3% vs -11.9%; P<0.05 for both comparisons). Incidence of a continuous gap in adherence of 90 days increased at twice the rate in the control groups vs the exposure group (+24.6% and +24.1 vs +11.7%; P<0.0001 for both comparisons).<br>• Cost had a definite impact on adherence.

There are no systematic reviews assessing the impact of formularies on prescribing appropriateness in the primary care setting and our review of literature databases found no RCTs. There are two controlled studies from the United Kingdom that evaluated the impact of limited formularies on prescribing variation. A formulary was introduced to 50 general practitioners from 11 family practices. The percentage of formulary medications prescribed and the number of different agents prescribed was measured on a yearly basis from 1992 – 1994 in these family practices (intervention) compared to all other practices in the county (control). The percentage

of formulary medications prescribed appropriately increased in three therapeutic categories: cardiovascular (7 – 12% above control), musculoskeletal (1 – 11% above control) and obstetrics and gynaecology (by 6 – 9% above control).[30] The number of different agents prescribed declined in three therapeutic categories: musculoskeletal (1 – 7% below control), nervous (7 – 12% below control) and nutrition and blood (15 – 21% below control).[30] Similarly, following the introduction of a formulary for NSAIDs to ten practices, the number of different NSAIDs used dropped significantly (14.30 to 13.10, p<0.04) while the percentage of the three most commonly used NSAIDs increased significantly (34.22 from 32.43, P<0.02) in one year.[31]

Several observational studies in the United States have found an association between mailing medications and improved medication adherence. Medicare Part D beneficiaries initiating oral anti-diabetic medications had significantly (P<0.001) better adherence through mail-order pharmacy (49.7%) compared to retail pharmacies (42.8%).[17] Similarly, patients who were part of a Kaiser Permanente Northern California diabetes registry who received a new antiglycemic, antihypertensive or lipid-lowering index medication were significantly more likely (P<0.001) to have good adherence by mail-order pharmacy (84.7%) than visiting pharmacies (76.9%).[19]

In summary, results of trials of different interventions that included providing free medications to different patient populations and observational studies suggest – but do not demonstrate - that our intervention will improve appropriate adherence. This trial is needed because the provision of a comprehensive set of medications to a general patient population has the potential to have a dramatic impact on healthcare in Canada but has not been studied. No trial has addressed cost-related non-adherence in a primary care population and no Canadian report or study has directly addressed the impact of medication costs on health outcomes.

1.5 How will the results of this trial be used?

The results of this project will help policy makers who are deliberating about what access Canadians should have to medications. Medications are currently provided to outpatients in other developed countries where healthcare services are publicly funded. In Canada, strong economic and social justice arguments have been made for providing medications without charge [1], but this has not yet happened. This study will provide empirical evidence related to prescribing patterns, medication adherence, health outcomes and costs, and will provide evidence in support of national pharmacare if the results are positive.

1.6 Describe any risks to the safety of participants involved in the trial.

Patients in the intervention group will have improved access to the medications on the list of essential medications but retain their usual access to all medications (i.e., they can choose to pay for medications that are not on the list). Thus access is not reduced for any medications.

In addition to the usual risks associated with all medications that will be experienced by participants in both the control usual care group and the intervention group, participants in the intervention group are at risk of being exposed to or experiencing:

i. Errors in switching to medications on the list of essential medications
ii. Dissatisfaction with the new medications
iii. Discontinuation effects of medications at the conclusion of the study

The risk of medication errors will be mitigated by having the pharmacist receiving the prescribed medication order review the electronic medical record which includes the history of medications previously prescribed. The pharmacist will use defined daily doses (DDD) defined by the WHO, which is a widely accepted standard for converting doses of equivalent

medications (these types of substitutions are currently routine for hospitalized patients).[32] The patients will also be contacted about the change in medications. Errors will thus only effect the medications taken by patients if they are made by prescribers and missed by both the pharmacist and the patient. Medication errors will be monitored by the Data and Safety Monitoring Board (DSMB).

In some cases, patients will be switched from brand name to generic products. Despite clear evidence that generics and brand name medications have similar clinical effects [33, 34], there is a perception by some that generics are inferior.[35] A RCT of "apparent" drug substitution (all pills were placebos) found that switching participants to placebos labeled as generic from branded placebos was associated with increased side effects such as dizziness, headache and dry mouth.[36] Medication adverse effects will be monitored by the DSMB.

At the conclusion of the study, participants in the intervention group will revert to their usual access to medications. In some cases participants will continue to access their medications while other participants will experience cost-related nonadherence after the conclusion of the trial. There are potential harms of stopping certain medications that have withdrawal symptoms (e.g antidepressants). Patients will be made aware of this possibility before they enroll in the study, before they start the medications and at the end of the trial. We will provide clinicians with instructions about how to manage patients who experience discontinuation effects, which usually resolve spontaneously. Discontinuation effects will be reported as medication adverse effects.

2. The Proposed Trial

2.1 What is the proposed trial design?

This will be an open label, parallel two arm, superiority, individually randomized controlled trial with 1:1 allocation.

2.2 What are the planned trial interventions?

The experimental intervention is conveniently providing essential medications to patients at no charge (see cleanmeds.ca for the list of medications). Both intervention patients and their prescribing clinicians will have access to the list of essential medications. Clinicians may prescribe new medications from the list of essential medications and patients may be switched to a listed medication (e.g from an unlisted ACE inhibitor to a listed one). Patients will receive listed medications at no charge. Patients may still be prescribed other medications and access them in the usual way (e.g. by paying for them).

Medications that need to be started in a timely fashion (e.g. antibiotics) will be available at the clinic. Long term medications will be dispensed by clinical pharmacists who have direct access to patient electronic medical records and prescriptions. These medications will be mailed with a default frequency of every 90 days, but alternative frequencies can be employed. Controlled substances (e.g. opioids, sedatives, and stimulants) will not be included in the intervention for safety reasons and patients will access them in the usual fashion.

After shipment, the pharmacist who will have access to interpretation services in 200 languages will call to counsel patients about their medications. Patients without a permanent address may choose an address (e.g. the clinic or a support centre) and those without a telephone may call the pharmacist for information about medications (e.g. from the clinic). Patients who are unable to communicate over the phone will be eligible for visits from the pharmacist.

Other potential prescribers outside of the clinic (e.g. consultants) will be faxed a letter with the list of essential medications and patients will be provided with a card with information about the list that can be shared with other providers. Both communications will include contact information for the study pharmacist.

The control condition is usual care with usual access to medications and dispensing.

2.3 What are the proposed practical arrangements for allocating participants to trial groups?

This is a double arm RCT at one urban and two rural sites. A randomization schedule for each site will be generated by computer to ensure allocation concealment. After a participant has provided informed consent, the site research coordinator will allocate the patient to one of the two groups using the allocation sequence from a computer generated random sequence.

2.4 What are the proposed methods for protecting against sources of bias?

The investigators and analysts will be blinded to treatment allocation to reduce ascertainment bias. Patients, clinicians and pharmacists must know the arm of allocation. An intention-to-treat analysis will be employed.

Contamination in this trial is unlikely for the long term prescription medications in this trial. These are the medications most commonly associated with non-adherence and they are thus the primary target of the trial. Multiple patients within one family will be excluded to prevent contamination within a family. Contamination is possible for the medications that need to be started in a timely fashion and thus they must be available at the clinics. The study team will audit mandatory stocking and dispensing records for medications stored on site to reduce contamination. Some prescribing contamination may occur in which a clinician prescribes medications from the list to control patients who have their usual access (i.e. patients will pay for the medications). Any contamination will tend to reduce the effect size (i.e., it will increase adherence in the control group and may reduce adherence in the intervention group). The low risk of contamination is one reason that we have decided to conduct an individual RCT rather than a cluster RCT which would involve a larger number of sites and be substantially more expensive.

Some patients may acquire public medication insurance during the follow-up period. All patients will be analyzed using the intention-to-treat method and the number of such patients in each arm will be reported.

2.5 What are the planned inclusion/exclusion criteria?

Inclusion criteria

- Self-reported cost-related medication non-adherence in last 12 months. We will use phrasing used in the Canadian Community Health Survey and similar surveys in other countries: "In the last twelve months, did you not fill a prescription or do anything to make a prescription last longer *because of the cost*?"[5, 37]
- Age 18 or older.

Exclusion criteria

- Valid public medication insurance (Ontario Drug Benefit) card.
- Family member living at same address of patients already enrolled.
- Joined practice within last 6 months.

We will exclude patients who are new to the practice as patients joining the practice in order to participate in the study is a potential source of bias and because some secondary outcomes require baseline assessments that will not be available for new patients.

We will not exclude patients who are eligible for public medication insurance but who do not have such coverage (e.g. patients who have lost or cannot access their benefit card).

We will not exclude patients with private medication insurance as long as they answer "yes" to the first question in the inclusion criteria. 7 % of Canadians who have medication coverage still report cost-related nonadherence.[5] Private insurance often requires some out of pocket expenses. Individuals who have private insurance and are not experiencing cost-related barriers to medication adherence will not be enrolled as they will not meet the inclusion criterion.

2.6 What is the proposed duration of the treatment period?

The treatment period will be for 12 months for each patient. (In a separate study, we hope to continue providing patients with these medications for free after the 12 months.)

2.7 What is the proposed frequency and duration of follow up?

The follow up period is identical to the treatment period (and so will also be 12 months for all participants). The 12 months of treatment and outcome assessment allows for the evaluation of clinical outcomes and cumulative costs adequately. Medication adherence and health outcomes data (blood pressure, HbA1c levels and cholesterol levels) will be collected during regularly scheduled appointments. Inappropriate prescribing, prescription records and costs will be reviewed from the electronic medical records.

2.8 What are the proposed primary and secondary outcome measures?

Primary outcome: Proportion (%) of patients who appropriately adhered to all prescribed treatments (i.e. were adherent to all prescriptions deemed appropriate).

Secondary outcome:

1. Proportion (%) of prescriptions that are appropriate.
2. Proportion (%) of prescriptions that are adhered to.
3. HbA1c levels in patients with diabetes (adjusted for baseline).
4. Blood pressure in hypertensive patients (adjusted for baseline).
5. LDL cholesterol levels in patients taking a statin (adjusted for baseline).
6. Per capita medication costs.

2.9 How will the outcome measures be measured at follow up?

We will use a combination of established methods for assessing the primary outcome: appropriate medication adherence, which requires assessment of both adherence and appropriateness [3].

There is no "gold standard" measure of medication adherence because more invasive measures of adherence can change actual adherence. We will employ questionnaires, chart reviews and electronic monitoring as recommended and previously used in trials[38]. Standard and validated medication adherence questions will be completed by patients over the telephone or during their regularly scheduled clinic visits between months 9 and 12 to determine the proportion of reported missed doses ("In the last week, how many times did you miss taking your regular medications?").[38] Self-reported non-adherence, taking into consideration the number of expected medication doses, greater than 80 % will be considered adherent (e.g. one or zero missed doses in one week for a medication taken once a day). While prone to memory bias as patients attempt to recall their adherence patterns[3], questionnaires are not as susceptible to social desirability bias as interviews due to anonymity[3, 38] and do not suffer from a drop in completion rates as a study progresses, as diaries do[39]. Chart reviews will be conducted by accessing electronic medical records (EMRs) to determine if long term medications are re-ordered when expected (i.e. for patients who are prescribed a three month supply of medication,

evidence of written prescriptions every three months would indicate adherence) in a blinded fashion. Prescriptions written within 18 days of the expected renewal date (20 % of typical renewal period of 90 days) will be classified as adherent. While susceptible to pill dumping, like other objective measures such as pill counts[3], EMR reviews do not depend on recall. Electronic monitoring is considered by many the best method for measuring adherence and is the only method that can practicably provide daily dosage information[40]. However, given the high costs involved only a small subset of patients in the study will be measured in this way[40]. The device chosen for this study is the Medication Event Monitoring Systems (MEMs) device, as it is the most widely used[40]. If bottle openings to take medications take place greater than 80% of the times expected, a patient will be classified as adherent. Self-reporting has been shown to overestimate adherence[3] while electronic monitoring is prone to underestimation[40].

This combination of three methods for assessing adherence will be more rigorous than the single methods used in previous trials. [29, 48, 49]

Adherence will be treated as a dichotomous variable. Participants deemed non-adherent by any of the three methods will be classified as non-adherent for the primary analysis. We will also report adherence rates by each of the three measures for the intervention and control groups. Prescriptions for medications intended to be taken on an "as needed" basis (e.g. analgesics, salbutamol) will be excluded from the adherence analysis.

Prescribing appropriateness will be assessed by blinded assessors with access to patient electronic medical records including clinician notes. Assessors will review all prescriptions written to patients in control and intervention arms using independently developed explicit patient level prescribing appropriateness indicators.[41] These indicators address the management of common chronic diseases such as heart disease, diabetes and asthma.[41] Prescribing appropriateness is a dichotomous variable and thus each prescription will be counted as appropriate or not appropriate.

The ***primary outcome of appropriate adherence*** will be determined by assessing the percentage of patients who were both adherent to all prescribed medications and received no inappropriate prescriptions.

Secondary outcomes

Adherence and appropriate prescribing will each also be secondary outcomes. For each, we will report the proportion of all prescriptions in each arm that meet the above criteria.

The mean change in HbA1c for patients with diabetes will be a secondary outcome. HbA1c levels will be mandatorily determined between 9 and 12 months after randomization in patients who had an HbA1c level measured no more than 6 months before randomization and who were prescribed a treatment for diabetes (e.g. metformin, gliclazide, or insulin). HbA1c is easy to assess, is routinely measured and is known to be associated with clinically important outcomes such as myocardial infarction and mortality[42].

The mean change in blood pressure for patients with hypertension or diabetes will be a secondary outcome. This will be assessed mandatorily between 9 and 12 months after randomization in patients who had a blood pressure reading recorded no more than 6 months before randomization and who are prescribed an antihypertensive treatment. Blood pressure is routinely measured and is known to be associated with clinically important outcomes such as myocardial infarction,[43] stroke[44] and mortality.[45]

The mean change in low density lipoprotein cholesterol (LDL) will be a secondary outcome. LDL will be determined mandatorily between 9 and 12 months after randomization in

patients who had an LDL level measured within 12 months of randomization and who are prescribed a statin. LDL levels are routinely measured and known to be associated with clinically important outcomes such as myocardial infarction, stroke and mortality [46]. Reductions in LDL by statins and other treatments reduce the risk of death based on multiple statin trials and preliminary results from the IMPROVE-IT trial (ezitimibe-simvastatin versus statin) presented in November of 2014 [47].

HbA1c, blood pressure and LDL will be assessed by audits of the EMR.

Patient and community engagement are an important and large part of this study and separate funding has been obtained from St Michael's Hospital and the Toronto Central Local Health Integration Network for engagement around the development of intervention and the selection of outcomes. Briefly, patient, clinician and pharmacist concerns and experiences with the dispensing model will be collected using questionnaires, focus groups and one-on-one interviews. In addition, focus groups and patient panels organized in collaboration before, during and after the study will provide feedback on the intervention.

An analysis of medication costs will be performed as described below.

2.10 Will health service research issues be addressed?

A cost analysis of per capita medication costs between control and intervention arms will be conducted. The analysis cannot be described in detail here due to space restrictions. Briefly, we will determine the mean medication costs per patient and compare this between the intervention and control groups. We will use these data to estimate the cost implications of widespread implementation of this model using Ontario prescribing data (for public assistance recipients) from the Institute of Clinical and Evaluative Sciences and Canadian prescribing data from IMS Brogan. We will also assess any implications of health outcome changes (including projected impact on clinical outcomes such as mortality) and changes in healthcare resource limitation. The team includes several health economists (see section 3.2).

2.11 What is the proposed sample size and justification?

Based on previous studies[29, 48, 49] we expected that between 40% and 60% of patients in the control group will be appropriately adherent. We expect that at least 90% of patients in the intervention group will be compliant (i.e. accept the free medications offered in the intervention). We believe that a 10% absolute improvement in appropriate adherence (in the presence of non-compliance) is the minimal difference that is clinically important. Assuming a Type I error probability of 5%, a sample size of 392 per group is required to have 80% power to detect a 10% absolute difference in adherence for any control group adherence values between 40% and 60%. No inflation for drop-out is applied since drop-outs will be considered non-adherent. Based on previous trials we expect the drop-out rate to be approximately 5 %.[29, 48, 49]

2.12 What is the planned recruitment rate? How will the recruitment be organized?

We project a recruitment rate of 75 patients per week based on a chart review and on completed RCTs.[29,48,49] Thus recruitment should require less than three months.

Participants will be recruited during routine primary care visits. Based on our chart review, primary care providers see at least 320 unique patients per week (approximately 4 per hour) and approximately 20 % of encounters involve a prescription for a medication. If 20 % of these 64 patients per week who receive a prescription medication are approached by an embedded study research assistant (32 approaches per week), and 20 % are eligible (i.e. report cost-related nonadherence), then 2-4 patients per clinician will be eligible to participate. If 50 %

of eligible patients agree to enroll in the study (to have a chance of receiving medications for free), each clinician will recruit approximately 1-2 patients per week.

There are a total of 50 full time equivalent clinicians at the three sites. Thus approximately 75 patients can be recruited each week.

This estimate is conservative as it assumes: that approaches will only take place in encounters involving a prescription medication while care providers may choose to approach patients at other encounters (e.g. a patient on a long term medication who has not presented for a prescription), a moderate approach rate, a low rate of self-reported cost-related non-adherence and a low rate of agreement to participate in a trial of receiving medications for free.

2.13 Are there likely to be any problems with compliance?

The primary outcome is medication adherence by patients. In this trial, "compliance" with the intervention refers to participants deciding to receive the essential medications without charge rather than taking the medications they were previously taking. We anticipate that few patients will choose to enroll in this study but then choose not to take *any* medications provided for free. Some participants may choose to take some medications from the list of essential medications and to also take medications that are not on the list (and this will not pose a problem for the trial). For the sample size calculation, we have assumed a compliance rate of 90 % for the intervention group.

2.14 What is the likely rate of loss to follow up?

We expect a low loss to follow up rate because participants are established patients of family practices. Medication adherence from written prescriptions, prescribing appropriateness and cost analyses can be assessed using information extracted directly from electronic medical records regardless of patient follow-up. The rate of being lost to follow up ranged from 5 to 16 % in the control group and between 5 and 11 % in the intervention group of previous trials.

Study	Control	Intervention	Reasons
Choudhry 2011	151/3010 (5.0 %)	133/2845 (4.7 %)	Participants lost insurance coverage
Farooq 2011	9/55 (16.4 %)	6/55 (10.9 %)	Drop out, died, discontinued intervention, withdrew consent
Thom 2013	50/1002 (5.0 %)	47/1002 (4.7 %)	Deaths, refused further participation, unable to contact, other

If a participant leaves the primary care practice, we will attempt to obtain the name of the new care provider and obtain the chart for review. If a participant has left the primary care practice and we are unable to contact them, we will censor the data from that participant after they have left the primary care practice.

2.15 How many centers will be involved?

The trial will be conducted in one urban family practice affiliated with St. Michael's Hospital in Toronto and two rural family practices in Ontario: Blind River and Manitoulin Island. All sites meet specifications including the use of the same electronic medical record system.

2.16 What is the proposed type of analyses?

Patient demographics will be summarized descriptively (e.g. means and SD or median IQR for continuous variables and frequency and percentages for categorical). Although randomization guarantees balance in the long-run, there is a chance of imbalances in any sample. The demographics will be reviewed for clinically important imbalances that may be adjusted for in a secondary analysis.

The primary analysis will be performed on an intention to treat basis. All patients can be included since drop-outs or withdrawals will be considered non-adherent. Appropriate adherence

will be compared using a chi-square test and the unadjusted treatment effect will be expressed as the absolute risk difference with 95% confidence interval. For the primary analysis a p-value of <0.05 will be sufficient to reject the null hypothesis of no difference. Adjusted analyses will employ logistic regression and the adjusted (conditional) treatment effect will be expressed as an odds ratio with 95% confidence interval.

The first two secondary outcomes are binary at the level of prescription within patient. This will be aggregated to the patient level and logistic regression will be used to compare groups. HbA1C, blood pressure and LDL cholesterol are all continuous and measured at baseline and 9-12 months. An analysis of covariance (ANCOVA) model will be used (via linear regression) to provide the estimates of treatment effect adjusted for baseline values. Since these are all secondary analyses which are supportive or exploratory, no corrections for multiple testing will be applied and only patients with data will be included. However the ITT approach is still followed as patients will remain allocated to their randomized groups irrespective of compliance.

2.17 What is the proposed frequency of analyses?

Analysis will occur at the completion of the study when all data are collected. No interim analyses are planned.

2.18 Are there any planned subgroup analyses?

We will determine if the effects on medication adherence and health outcomes differ by age and sex.

2.19 Has any pilot study been carried out using this design?

The intervention is complex and substantial resources have been dedicated to its development. We have verified the feasibility of chart reviews similar to those that will be used for outcome ascertainment. We believe that a full trial of the intervention is warranted (see *section 1.1*).

3. Trial Management

3.1 What are the arrangements for the day to day management of the trial?

The study will be coordinated from St Michael's Hospital in Toronto. The clinical sites will have site research coordinators who will be responsible for recruitment, training of clinicians and patients, and data collection for the duration of the project. They will liaise with a clinical research coordinator in Toronto who will review their data and provide further instructions as required. The lead clinical research coordinator will also be responsible for liaising with the pharmacist, REB board, principal investigators and safety committees. The research team will receive trial implementation assistance from the Applied Health Research Centre (AHRC) who will advise on trial coordination, site training, site start-up/activation, document management, supply management, database development, data management and statistical analysis. The AHRC has experience managing more than 50 multi-site, national and international studies including 15 CIHR-funded multi-centre trials and has grown to be one of the largest academic research organizations in Canada.

Study data and patient questionnaires will be entered and maintained on a secure password protected database developed using Medidata RAVE (www.mdsol.com) and will be accessible via the internet for data entry purposes. Quality and completeness of data entry will be reviewed as soon as possible after data entry, within 5 business days of data entry for the first 5 participants randomized at each site, and within 15 days of data entry thereafter. Corrections or

changes in the data management system will be tracked with the retention of the original data and the corrected data with the date of data entry and submitting personnel.

3.2 What will be the role of each principal applicant and co-applicant proposed?

The co-principal investigator Andreas Laupacis (Canada Research Chair in Health Policy and Citizen Engagement and Executive Director, Li Ka Shing Knowledge Institute) is mentoring the principal applicant Nav Persaud through a CIHR RCT training grant to develop this trial.

AL and NP will together oversee all aspects of the project with input from all co-investigators. Some relevant experiences of the co-investigators are briefly highlighted here. Muhammad Mamdani (Director of the Applied Health Research Centre), Stephen Hwang (Scientist at the Centre for Research on Inner City Health), and Andrew Pinto (Associate Scientist, MSc Health Policy, Planning and Financing) have experience conducting RCTs of complex health interventions. Kevin Thorpe (Assistant Professor) is a biostatistician with experience from dozens of RCTs. Richard Glazier (Senior Scientist and Program Lead at the Institute for Clinical Evaluative Sciences), Steve Morgan (PhD in Economics) and Braden Manns (Svare Professor in Health Economics) have experience relevant to the health economics evaluation. Michael Law (PhD in Health Policy) and Dr. Paul Oh (Scientist at the Toronto Rehabilitation Institute) have expertise in pharmaceutical policy that is relevant both to the study design and the knowledge translation plan. Danielle Martin (Masters of Public Policy) and Andrew Boozary (Masters of Public Policy) have experience communicating scientific findings to policymakers.

3.3 Describe the trial steering committee and any data safety and monitoring committee.

The trial steering committee will include the two study principal investigators (NP and AL), a patient representative, a pharmacist, and a biostatistician.

The primary purpose of the data safety and monitoring committee will be to ensure medication errors are properly addressed. Since we expect medication errors to be rare, each medication error will be reported to the DSMB immediately. The DSMB will make recommendations to the research team about how to mitigate the harm from the medication error and how to prevent future similar errors. The DSMB will have the power to recommend that the trial be stopped if there is an excess of medication errors or if identified errors are not appropriately handled.

The chair of the DSMB will be Dr Dee Mangin who is a family physician and research scientist with experience conducting clinical trials in primary care related to prescribing appropriateness.

References

1. Gagnon, M.-A., *A Roadmap to a Rational Pharmacare Policy in Canada*. 2014, School of Public Policy & Administration, Carleton University.
2. Osterberg, L. and T. Blaschke, *Adherence to medication.* N Engl J Med, 2005. **353**(5): p. 487-97.
3. Lehmann, A., et al., *Assessing medication adherence: options to consider.* Int J Clin Pharm, 2014. **36**(1): p. 55-69.
4. Tamblyn, R., et al., *The incidence and determinants of primary nonadherence with prescribed medication in primary care: a cohort study.* Ann Intern Med, 2014. **160**(7): p. 441-50.
5. Law, M.R., et al., *The effect of cost on adherence to prescription medications in Canada.* Cmaj, 2012. **184**(3): p. 297-302.
6. Choudhry, N.K., et al., *Cost-effectiveness of providing full drug coverage to increase medication adherence in post-myocardial infarction Medicare beneficiaries.* Circulation, 2008. **117**(10): p. 1261-8.
7. *Ontario Public Drug Programs - Formulary - Drugs Funded by Ontario Drug Benefit (ODB) Program*. 2014 [cited 2014 8/25/2014]; Available from: http://www.health.gov.on.ca/en/pro/programs/drugs/odbf_mn.aspx.
8. Howard, M., et al., *Prescribing of potentially inappropriate medications to elderly people.* Fam Pract, 2004. **21**(3): p. 244-7.
9. McGettigan, P. and D. Henry, *Use of non-steroidal anti-inflammatory drugs that elevate cardiovascular risk: an examination of sales and essential medicines lists in low-, middle-, and high-income countries.* PLoS Med, 2013. **10**(2): p. e1001388.
10. Bhala, N., et al., *Vascular and upper gastrointestinal effects of non-steroidal anti-inflammatory drugs: meta-analyses of individual participant data from randomised trials.* Lancet, 2013. **382**(9894): p. 769-79.
11. CIHI, *National Health Expenditure Trends, 1975 to 2014*. 2014, CIHI.
12. Gustafsson, L.L., et al., *The 'wise list'- a comprehensive concept to select, communicate and achieve adherence to recommendations of essential drugs in ambulatory care in Stockholm.* Basic & Clinical Pharmacology & Toxicology, 2011. **108**(4): p. 224-33.
13. OECD. *Pharmaceutical expenditure per capita*. 2014 2/20/2015]; Available from: http://www.oecd-ilibrary.org/social-issues-migration-health/pharmaceutical-expenditure-per-capita_pharmexpcap-table-en.
14. Morgan, S.G., J.R. Daw, and M.R. Law, *Rethinking pharmacare in Canada*. 2013: CD Howe Institute.
15. Gagnon, M.D. and G. Hebert, *The Economic Case for Universal Pharmacare: Costs and Benefits of Publicly Funded Drug Coverage for all Canadians*. 2010.
16. Wu, C., C.M. Bell, and W.P. Wodchis, *Incidence and economic burden of adverse drug reactions among elderly patients in Ontario emergency departments: a retrospective study.* Drug Saf, 2012. **35**(9): p. 769-81.
17. Zhang, L., et al., *Mail-order pharmacy use and medication adherence among Medicare Part D beneficiaries with diabetes.* Journal of Medical Economics, 2011. **14**(5): p. 562-7.
18. Adams, A.S., et al., *Health system factors and antihypertensive adherence in a racially and ethnically diverse cohort of new users.* JAMA Internal Medicine, 2013. **173**(1): p. 54-61.
19. Duru, O.K., et al., *Mail-order pharmacy use and adherence to diabetes-related medications.* American Journal of Managed Care, 2010. **16**(1): p. 33-40.

20. Johnsrud, M., K.A. Lawson, and M.D. Shepherd, *Comparison of mail-order with community pharmacy in plan sponsor cost and member cost in two large pharmacy benefit plans.* Journal of Managed Care Pharmacy, 2007. **13**(2): p. 122-34.
21. Visaria, J., et al., *Comparing the cost of community pharmacy and mail-order pharmacy in a US retirement system.* International Journal of Health Planning & Management, 2012. **27**(1): p. e41-50.
22. Schmittdiel, J.A., et al., *Safety and effectiveness of mail order pharmacy use in diabetes.* American Journal of Managed Care, 2013. **19**(11): p. 882-7.
23. Schmittdiel, J.A., et al., *The comparative effectiveness of mail order pharmacy use vs. local pharmacy use on LDL-C control in new statin users.* Journal of General Internal Medicine, 2011. **26**(12): p. 1396-402.
24. Beardon, P.H., et al., *Introducing a drug formulary to general practice--effects on practice prescribing costs.* J R Coll Gen Pract, 1987. **37**(300): p. 305-7.
25. Romanow, R.J., *Building on Values: The Future of Health Care in Canada - Final Report*. 2002, Government of Canada.
26. Kirby, M.J. and M. LeBreton, *The Health of Canadians - The Federal Role - Final Report - Volume Six: Recommendations for Reform*. 2002, The Standing Senate Committee on Social Affairs, Science and Technology.
27. Hoskins, E., *Eric Hoskins: The time for national pharmacare has come*, in *Toronto Star*. 2014: Toronto.
28. Nieuwlaat, R., et al., *Interventions for enhancing medication adherence.* Cochrane Database Syst Rev, 2014. **11**: p. Cd000011.
29. Choudhry, N.K., et al., *Full coverage for preventive medications after myocardial infarction.* New England Journal of Medicine, 2011. **365**(22): p. 2088-97.
30. Hill-Smith, I., *Sharing resources to create a district drug formulary: a countywide controlled trial.* British Journal of General Practice, 1996. **46**(406): p. 271-75.
31. Avery, A.J., et al., *Do prescribing formularies help GPs prescribe from a narrower range of drugs? A controlled trial of the introduction of prescribing formularies for NSAIDs.* British Journal of General Practice, 1997. **47**(425): p. 810-4.
32. WHO. *DDD - Definitions and general considerations*. 2009 2009/12/17 2015/2/13]; Available from: http://www.whocc.no/ddd/definition_and_general_considera/.
33. Kesselheim, A.S., et al., *Clinical equivalence of generic and brand-name drugs used in cardiovascular disease: a systematic review and meta-analysis.* Jama, 2008. **300**(21): p. 2514-26.
34. Kesselheim, A.S., et al., *Seizure outcomes following the use of generic versus brand-name antiepileptic drugs: a systematic review and meta-analysis.* Drugs, 2010. **70**(5): p. 605-21.
35. Guberman, A. and C. Corman, *Generic substitution for brand name antiepileptic drugs: a survey.* Can J Neurol Sci, 2000. **27**(1): p. 37-43.
36. Faasse, K., et al., *The effect of an apparent change to a branded or generic medication on drug effectiveness and side effects.* Psychosomatic Medicine, 2013. **75**(1): p. 90-6.
37. Canada, H. *Canadian Community Health Survey*. 2012 2012/04/23 2015/2/13]; Available from: http://www.hc-sc.gc.ca/fn-an/surveill/nutrition/commun/index-eng.php.
38. Garber, M.C., et al., *The concordance of self-report with other measures of medication adherence: a summary of the literature.* Med Care, 2004. **42**(7): p. 649-52.
39. Butz, A.M., et al., *Monitoring nebulizer use in children: comparison of electronic and asthma diary data.* Ann Allergy Asthma Immunol, 2005. **94**(3): p. 360-5.
40. Cook, P., et al., *Practical and analytic issues in the electronic assessment of adherence.* West J Nurs Res, 2012. **34**(5): p. 598-620.

41. Farooq, S., et al., *Schizophrenia medication adherence in a resource-poor setting: randomised controlled trial of supervised treatment in out-patients for schizophrenia (STOPS).* Br J Psychiatry, 2011. **199**(6): p. 467-72.
42. Thom, S., et al., *Effects of a fixed-dose combination strategy on adherence and risk factors in patients with or at high risk of CVD: the UMPIRE randomized clinical trial.* Jama, 2013. **310**(9): p. 918-29.
43. Fernandez Urrusuno, R., et al., *Compliance with quality prescribing indicators in terms of their relationship to financial incentives.* European Journal of Clinical Pharmacology, 2013. **69**(10): p. 1845-53.
44. Macisaac, R.J. and G. Jerums, *Intensive glucose control and cardiovascular outcomes in type 2 diabetes.* Heart, Lung & Circulation, 2011. **20**(10): p. 647-54.
45. Papademetriou, V., *From Hypertension to Heart Failure.* The Journal of Clinical Hypertension, 2004. **6**: p. 14-17.
46. Nagai, M. and K. Kario, *Visit-to-visit blood pressure variability, silent cerebral injury, and risk of stroke.* Am J Hypertens, 2013. **26**(12): p. 1369-76.
47. Briasoulis, A., et al., *Effects of antihypertensive treatment in patients over 65 years of age: a meta-analysis of randomised controlled studies.* Heart, 2014. **100**(4): p. 317-23.
48. Stone, N.J., et al., *Treatment of blood cholesterol to reduce atherosclerotic cardiovascular disease risk in adults: synopsis of the 2013 American College of Cardiology/American Heart Association cholesterol guideline.* Ann Intern Med, 2014. **160**(5): p. 339-43.
49. Blazing, M.A., et al., *Evaluating cardiovascular event reduction with ezetimibe as an adjunct to simvastatin in 18,144 patients after acute coronary syndromes: final baseline characteristics of the IMPROVE-IT study population.* Am Heart J, 2014. **168**(2): p. 205-12.e1.

CLEAN Meds RCT: Carefully seLected and Easily Accessible at No charge Medications

Canada is the only high income country where health care services are publicly funded for the general population but medications are not.[1] We propose an RCT of providing carefully selected, essential medications at no charge (compared with usual medication access) to primary care patients. The impact of this intervention on appropriate medication adherence, valid surrogate health outcomes and costs will be determined:

Population – Adult primary care patients who report cost-related non-adherence to medications
Intervention – Free and convenient access to a carefully selected set of essential medications with otherwise usual care (n = 392)
Control – Usual access to medications and usual care (n = 392)
Outcome – *Primary*: Appropriate adherence to medications over 24 months. Secondary: HbA1c levels (in patients with diabetes); blood pressure (in hypertensive patients); cholesterol (in patients with dyslipidemia); average per person medication costs.

1. The Need for a Trial

1.1 What is the problem to be addressed?

A large number of different medications are prescribed in Canada and a much larger number of Canadians do not take prescribed medications because of the cost. The intervention in this trial – the free provision of a carefully selected set of medications – has the potential to address both medication selection and medication adherence.

Medication adherence is an important determinant of health outcomes.[2] Approximately one half of treatments for chronic disease are not taken as directed.[3] Cost is one of several important causes of poor adherence and it is particularly important for vulnerable people.[4, 5] At least 2.4 million adult Canadians report cost related nonadherence to medications.[5] 31 % of prescriptions in Quebec are not filled and those of highest cost are least likely to be filled.[4] Providing full drug coverage may improve medication adherence and clinical outcomes, particularly for vulnerable individuals and those with chronic diseases.[6]

The size of many Canadian formularies such as the Ontario Drug Benefit Formulary, which contains over 3800 medications[7], makes it difficult for clinicians to know the safest and most effective medications to prescribe. This may partially explain poor medication selection. For example 16% of older Canadian primary care patients receive at least one potentially inappropriate medication.[8] 17% of NSAID prescriptions are for diclofenac[9] even though it is associated with additional vascular risk compared to other NSAIDs such as naproxen and has no advantage.[10] Inappropriate prescribing has been linked to excess morbidity and hospitalization.[8]

Approximately 12 billion dollars are spent each year by provincial and territorial governments on medications in Canada.[11] From 2000 to 2010, the annual average per capita growth in prescription expenditures in Canada was approximately 4%.[1] In contrast, comparable countries such as the United Kingdom only experienced growth approximating 1%.[1] The large number of medications in formularies may contribute to the high prices of medications in Canada. Medication costs are lower in countries such as Sweden with limited formularies (similar to the list of essential medications that will be employed in this study).[12] Per capita medication costs in 2011 for Canada and Sweden were $771 and $477,

respectively.[13] Multiple national reports over several decades have concluded that a national pharmacare system would save billions of dollars annually from a societal perspective[1, 14, 15] yet provincial decision makers have cited cost considerations in deciding against making large formularies containing thousands of medications[7] available to all residents.[1] The use of a carefully selected set of medications might be dramatically less expensive, yet still provide the general population with access to the majority of medications they need.

Access to medications is dependent on local pharmacies. In Canada, the current dispersion of dispensing over multiple pharmacies is associated with a greater risk of severe adverse drug reactions in the elderly.[16] This may be due to incomplete communication among prescribers and dispensing pharmacists.[16] The use of an integrated prescriber-pharmacist medication dispensing system has showed great promise. In this system, the clinician and pharmacist both have access to the electronic medical record where prescriptions are entered. The pharmacist dispenses medications by mail to the patient and provides counseling over the telephone. Observational studies have demonstrated that medication adherence and efficiency are greater when patients are mailed prescription medications.[17-21] Observational studies have also found an association between mailing prescription medications and fewer emergency room visits[22] and better management of dyslipidemia.[23]

An intervention that combines three critical elements – free access, a carefully selected set of medications, and centralized distribution and mailing – is a logical focus for a randomized controlled trial that would provide important information for policy makers. Other short evidence-based lists of medications have been developed, but trials have demonstrated that merely educating clinicians about medication lists does not change practice or the medications patients actually receive.[24] In this intervention, prescribing will be driven to the selected medications because patients will have access to these medications at no expense. It would be impractical and expensive to give patients access to thousands of different medications at no charge; a short list of medications couples naturally with centralized medication distribution for this trial and is easily scalable if the trial results are positive. The individual components of the intervention could also be trialed if the results of this trial are positive.

1.2 What are the principal research questions to be addressed?

Primary question: Does providing convenient access to a carefully selected set of essential medications for free to adult primary care patients increase appropriate medication adherence?

Secondary questions:

Does providing convenient access to a carefully selected set of essential medications for free to adult primary care patients:

1) Increase appropriate prescribing?
2) Increase treatment adherence (regardless of whether prescriptions are appropriate)?
3) Improve HbA1c levels in patients with type 2 diabetes on oral hypoglycemic medications?
4) Improve blood pressure control in hypertensive patients on antihypertensive medications?
5) Improve LDL cholesterol levels in patients taking a statin?
6) Reduce per capita medication costs?

1.3 Why is a trial needed now?

Over the past several decades, multiple reports have called for national Canada-wide pharmacare or a publicly funded medication system that incorporates a national formulary.[1, 14, 25, 26] Romanow's report concluded that a unified formulary is necessary for collective bargaining

efforts because some provinces and territories are too small to individually negotiate prices, are subject to intense lobbying efforts from the pharmaceutical industry, and are often pressured to make poor decisions based on decisions made by other provinces.[25] A national formulary has been argued to be necessary to improve access, reduce costs, improve practice and to allow the implementation of universal Canadian pharmacare. [1] Yet no such system exists in Canada.

Some health system leaders have recently expressed openness to policy change. In December of 2014 the Ontario Minister of Health wrote in a newspaper op-ed that he "put the issue of national pharmacare on the front burner at the meeting with [his] provincial, territorial, and federal colleagues" and that he "was encouraged by federal Health Minister Rona Ambrose's commitment after raising the need for a national pharmacare program."[27] The results of this trial may inform pharmacare policy discussions and they will be communicated directly to decision makers provincially and federally.

1.4 Give references to any relevant systematic review and discuss the need for your trial.

A recent Cochrane systematic review on interventions for enhancing medication adherence included one RCT that examined the impact of providing full coverage for medications after a myocardial infarction.[28] In the MI FREEE trial, the absolute adherence for all patients in the full coverage intervention group (43.9 ± 33.7%) was 5.4% (95% CI, 3.6–7.2%; P<0.001) higher compared to the usual coverage control group (38.9 ± 32.7%).[29] There was no significant improvement in the primary composite clinical outcome of the rate of first readmission for a major vascular event or coronary revascularization (17.6 intervention versus 18.8 control; HR, 0.93; 95% CI, 0.82 to 1.04; P=0.21).[29] However, secondary clinical outcomes of rates of total major vascular events or revascularization (21.5 intervention versus 23.3 control; HR, 0.89; 95% CI, 0.90 to 0.99; P=0.03) and rates of first major vascular event (11.0 intervention versus 12.8 control; HR, 0.86; 95% CI, 0.74 to 0.99; P=0.03) were significantly reduced in the full coverage group.[29] The table below explains how our trial differs from the MI FREEE trial:

	**MI FREEE**	**Our trial**
Setting	United States, hospitalized patients	Canada, outpatient
Patients	Patients hospitalized for MI	Primary care patients who report cost-related non-adherence
Intervention	Coverage of medications that would ordinarily be prescribed	Provision of only a carefully selected set of medications for free
Medication classes	Cardiovascular only	Almost all
Randomization methodology	Cluster, plan level	Individual patient
Medication adherence methodology	Medication possession ratio	Patient self-report, electronic monitoring, prescription timing
Clinical outcomes	Vascular events and other cardiovascular outcomes	HbA1c levels, blood pressure, LDL

Our review of the literature and registries identified no other RCTs assessing the impact on adherence of providing medications at no charge. The table below summarizes the most relevant studies where the intervention or exposure included access to medications for free. In all studies, the intervention differs from the control condition in ways other than free access to medications, and participants in the control groups had access to medications without charge.

	**Thom (2013)**	**Farooq (2011)**	**Doshi (2009)**
Design	RCT	RCT	Cohort
Setting	England, Ireland, Netherlands and India	Pakistan	USA

Population	Adults at high risk for cardiovascular disease	Adults with schizophrenia and schizoaffective disorders	Adults with an indication for lipid lowering treatment
Intervention or Exposure	Fixed dose combination pill provided for free.<br>N=1,002	Medication adherence counselling for participants and primary care giver with treatment administration supervised directly by care giver and medications free of charge.<br>N= 55	Usual care with medications free of charge.<br>N= 495
Control	Usual care with the *option* to receive usual medications for free.<br>N= 1,002	Usual care with the *option* to receive usual medications for free.<br>N= 55	Increased medication copayments but medications *not* free of charge.<br>N= 5,109
Outcome	Adherence to treatment (defined as the number of days medications were taken in the week prior to visit via self-reporting during visits) and changes in SBP and LDL-C from baseline at 12 months.	Adherence to the treatment (complete adherence defined as participants always taking medication as prescribed without any break during the assessment period via patient interview and pill counts) and changes in symptoms (Positive and Negative Syndrome Scale for Schizophrenia -PANSS) and functional (Global Assessment of Functioning -GAF) scores from baseline at 12 months.	Adherence to treatment defined as the proportion of days covered (the number of days with lipid-lowering drug supply on hand divided by the number of days in the specified time period) with lipid-lowering medications >=80% at 24 months.
Results	• Intervention group had better adherence and were more likely to adhere vs control group (829 (86%) vs 621 (65%); Relative Risk of being adherent, 1.33; 95% CI,1.26, 1.41; P<0.001)<br>• Interventional group had better clinical outcomes than the control group; reductions in SBP (−2.6mmHg; 95%CI, −4.0 to −1.1mmHg; P<0.001) and LDL-C (−4.2mg/dL; 95%CI, −6.6, −1.9mg/dL; P<0.001)	• Intervention group had better adherence and were more likely to adhere than control group (complete adherence: 37 (67.3%) vs 25 (45.5%); Relative Risk of being adherent 1.59; 95% CI, 1.03, 2.53; P<0.02).<br>• Interventional group had significant improvement in symptoms (PANSS Total scores: time effect P=0.017, between participant effect P=0.003 and PANSS Positive score: time effect P=0.011, between participants effect; P=0.003)	• Control group had significantly decreased adherence compared to the exposure group (-19.2% and -19.3% vs -11.9%; P<0.05 for both comparisons). Incidence of a continuous gap in adherence of 90 days increased at twice the rate in the control groups vs the exposure group (+24.6% and +24.1 vs +11.7%; P<0.0001 for both comparisons).<br>• Cost had a definite impact on adherence.

There are no systematic reviews assessing the impact of formularies on prescribing appropriateness in the primary care setting and our review of literature databases found no RCTs. There are two controlled studies from the United Kingdom that evaluated the impact of limited formularies on prescribing variation. A formulary was introduced to 50 general practitioners from 11 family practices. The percentage of formulary medications prescribed and the number of different agents prescribed was measured on a yearly basis from 1992 – 1994 in these family practices (intervention) compared to all other practices in the county (control). The percentage

of formulary medications prescribed appropriately increased in three therapeutic categories: cardiovascular (7 – 12% above control), musculoskeletal (1 – 11% above control) and obstetrics and gynaecology (by 6 – 9% above control).[30] The number of different agents prescribed declined in three therapeutic categories: musculoskeletal (1 – 7% below control), nervous (7 – 12% below control) and nutrition and blood (15 – 21% below control).[30] Similarly, following the introduction of a formulary for NSAIDs to ten practices, the number of different NSAIDs used dropped significantly (14.30 to 13.10, $p<0.04$) while the percentage of the three most commonly used NSAIDs increased significantly (34.22 from 32.43, $P<0.02$) in one year.[31]

Several observational studies in the United States have found an association between mailing medications and improved medication adherence. Medicare Part D beneficiaries initiating oral anti-diabetic medications had significantly ($P<0.001$) better adherence through mail-order pharmacy (49.7%) compared to retail pharmacies (42.8%).[17] Similarly, patients who were part of a Kaiser Permanente Northern California diabetes registry who received a new antiglycemic, antihypertensive or lipid-lowering index medication were significantly more likely ($P<0.001$) to have good adherence by mail-order pharmacy (84.7%) than visiting pharmacies (76.9%).[19]

In summary, results of trials of different interventions that included providing free medications to different patient populations and observational studies suggest – but do not demonstrate - that our intervention will improve appropriate adherence. This trial is needed because the provision of a comprehensive set of medications to a general patient population has the potential to have a dramatic impact on healthcare in Canada but has not been studied. No trial has addressed cost-related non-adherence in a primary care population and no Canadian report or study has directly addressed the impact of medication costs on health outcomes.

1.5 How will the results of this trial be used?

The results of this project will help policy makers who are deliberating about what access Canadians should have to medications. Medications are currently provided to outpatients in other developed countries where healthcare services are publicly funded. In Canada, strong economic and social justice arguments have been made for providing medications without charge [1], but this has not yet happened. This study will provide empirical evidence related to prescribing patterns, medication adherence, health outcomes and costs, and will provide evidence in support of national pharmacare if the results are positive.

1.6 Describe any risks to the safety of participants involved in the trial.

Patients in the intervention group will have improved access to the medications on the list of essential medications but retain their usual access to all medications (i.e., they can choose to pay for medications that are not on the list). Thus access is not reduced for any medications.

In addition to the usual risks associated with all medications that will be experienced by participants in both the control usual care group and the intervention group, participants in the intervention group are at risk of being exposed to or experiencing:

i. Errors in switching to medications on the list of essential medications
ii. Dissatisfaction with the new medications
iii. Discontinuation effects of medications at the conclusion of the study

The risk of medication errors will be mitigated by having the pharmacist receiving the prescribed medication order review the electronic medical record which includes the history of medications previously prescribed. The pharmacist will use defined daily doses (DDD) defined by the WHO, which is a widely accepted standard for converting doses of equivalent

medications (these types of substitutions are currently routine for hospitalized patients).[32] The patients will also be contacted about the change in medications. Errors will thus only effect the medications taken by patients if they are made by prescribers and missed by both the pharmacist and the patient. Medication errors will be monitored by the Data and Safety Monitoring Board (DSMB).

In some cases, patients will be switched from brand name to generic products. Despite clear evidence that generics and brand name medications have similar clinical effects [33, 34], there is a perception by some that generics are inferior.[35] A RCT of "apparent" drug substitution (all pills were placebos) found that switching participants to placebos labeled as generic from branded placebos was associated with increased side effects such as dizziness, headache and dry mouth.[36] Medication adverse effects will be monitored by the DSMB.

At the conclusion of the study, participants in the intervention group will revert to their usual access to medications. In some cases participants will continue to access their medications while other participants will experience cost-related nonadherence after the conclusion of the trial. There are potential harms of stopping certain medications that have withdrawal symptoms (e.g antidepressants). Patients will be made aware of this possibility before they enroll in the study, before they start the medications and at the end of the trial. We will provide clinicians with instructions about how to manage patients who experience discontinuation effects, which usually resolve spontaneously. Discontinuation effects will be reported as medication adverse effects.

2. The Proposed Trial

2.1 What is the proposed trial design?

This will be an open label, parallel two arm, superiority, individually randomized controlled trial with 1:1 allocation.

2.2 What are the planned trial interventions?

The experimental intervention is conveniently providing essential medications to patients at no charge (see cleanmeds.ca for the list of medications). Both intervention patients and their prescribing clinicians will have access to the list of essential medications. Clinicians may prescribe new medications from the list of essential medications and patients may be switched to a listed medication (e.g from an unlisted ACE inhibitor to a listed one). Patients will receive listed medications at no charge. Patients may still be prescribed other medications and access them in the usual way (e.g. by paying for them). All prescriptions must be entered into the EMR by clinicians.

Medications that need to be started in a timely fashion (e.g. antibiotics) will be available at the clinic. Long term medications will be dispensed by a clinical pharmacist who has direct access to patient electronic medical records and prescriptions. If the pharmacist disagrees or requires clarification they must contact the clinician before proceeding to dispense the medication by mail. The study pharmacist will have access to EMR records of all three sites and medications will be mailed from the partner pharmacy in Toronto. Where necessary, medications can be mailed from partner pharmacies of the rural sites with coordination from the study pharmacist. These medications will be mailed with a default frequency of every 90 days, but alternative frequencies can be employed. At the discretion of the trial participant's clinician and pharmacist the mailing frequency may be adjusted. The patient's needs and medication will govern adjustments. For example, medications with a short shelf life may need to be mailed out more

frequently (ex: once a month). Controlled substances (e.g. opioids, sedatives, and stimulants) will not be included in the intervention for safety reasons and patients will access them in the usual fashion. Medications that cannot be mailed, must be picked up at the clinic or community partner pharmacies of the three sites.
After shipment, the pharmacist who will have access to interpretation services in 200 languages will call to counsel patients about their medications. For medications that cannot be counseled over telephone, these will be provided in partner pharmacies or clinic where pharmacists will counsel the patients in their use (e.g. IUDs, inhalers, insulin injections). Patients without a permanent address may choose an address (e.g. the clinic or a support centre) and those without a telephone may call the pharmacist for information about medications (e.g. from the clinic). Patients who are unable to communicate over the phone will be eligible for visits from the pharmacist. Home visits will be conducted in rural sites by local family health team staff in line with usual practice.

Other potential prescribers outside of the clinic (e.g. consultants) will be faxed a letter with the list of essential medications and patients will be provided with a card with information about the list that can be shared with other providers. Both communications will include contact information for the study pharmacist.

The control condition is usual care with usual access to medications and dispensing.

2.3 What are the proposed practical arrangements for allocating participants to trial groups?

This is a double arm RCT at one urban and two rural sites. A randomization schedule for each site will be generated by computer to ensure allocation concealment. After a participant has provided informed consent, the site research coordinator will allocate the patient to one of the two groups using the allocation sequence from a computer generated random sequence.

2.4 What are the proposed methods for protecting against sources of bias?

The investigators and analysts will be blinded to treatment allocation to reduce ascertainment bias. Patients, clinicians and pharmacists must know the arm of allocation. An intention-to-treat analysis will be employed.

Contamination in this trial is unlikely for the long term prescription medications in this trial. These are the medications most commonly associated with non-adherence and they are thus the primary target of the trial. Multiple patients within one family will be excluded to prevent contamination within a family. Contamination is possible for the medications that need to be started in a timely fashion and thus they must be available at the clinics. The study team will audit mandatory stocking and dispensing records for medications stored on site to reduce contamination. Some prescribing contamination may occur in which a clinician prescribes medications from the list to control patients who have their usual access (i.e. patients will pay for the medications). Any contamination will tend to reduce the effect size (i.e., it will increase adherence in the control group and may reduce adherence in the intervention group). The low risk of contamination is one reason that we have decided to conduct an individual RCT rather than a cluster RCT which would involve a larger number of sites and be substantially more expensive.

Some patients may acquire public medication insurance during the follow-up period. All patients will be analyzed using the intention-to-treat method and the number of such patients in each arm will be reported.

2.5 What are the planned inclusion/exclusion criteria?

Inclusion criteria

- Self-reported cost-related medication non-adherence in last 12 months. We will use phrasing used in the Canadian Community Health Survey and similar surveys in other countries: "In the last twelve months, did you not fill a prescription or do anything to make a prescription last longer *because of the cost*?"[5, 37]
- Age 18 or older.

Exclusion criteria

- Valid public medication insurance (Ontario Drug Benefit) card.
- Family member living at same address of patients already enrolled.
- Joined practice within last 6 months.

We will exclude patients who are new to the practice as patients joining the practice in order to participate in the study is a potential source of bias and because some secondary outcomes require baseline assessments that will not be available for new patients.

We will not exclude patients who are eligible for public medication insurance but who do not have such coverage (e.g. patients who have lost or cannot access their benefit card).

We will not exclude patients with private medication insurance as long as they answer "yes" to the first question in the inclusion criteria. 7 % of Canadians who have medication coverage still report cost-related nonadherence.[5] Private insurance often requires some out of pocket expenses. Individuals who have private insurance and are not experiencing cost-related barriers to medication adherence will not be enrolled as they will not meet the inclusion criterion.

2.6 What is the proposed duration of the treatment period?

The treatment period will be for 24 months for each patient.

2.7 What is the proposed frequency and duration of follow up?

The follow up period is identical to the treatment period (and so will also be 24 months for all participants). The 24 months of treatment and outcome assessment allows for the evaluation of clinical outcomes and cumulative costs adequately. Medication adherence and health outcomes data (blood pressure, HbA1c levels and cholesterol levels) will be collected during regularly scheduled appointments. Inappropriate prescribing, prescription records and costs will be reviewed from the electronic medical records.

2.8 What are the proposed primary and secondary outcome measures?

Primary outcome: Proportion (%) of patients who appropriately adhered to all prescribed treatments (i.e. were adherent to all prescriptions deemed appropriate).

Secondary outcome:

1. Proportion (%) of prescriptions that are appropriate.
2. Proportion (%) of prescriptions that are adhered to.
3. HbA1c levels in patients with diabetes (adjusted for baseline).
4. Blood pressure in hypertensive patients (adjusted for baseline).
5. LDL cholesterol levels in patients taking a statin (adjusted for baseline).
6. Per capita medication costs.

2.9 How will the outcome measures be measured at follow up?

We will use a combination of established methods for assessing the primary outcome: appropriate medication adherence, which requires assessment of both adherence and appropriateness [3]. Participants will be asked to complete a survey on the telephone between 9 and 18 months and between 21 and 30 months after randomization. If patients are not reached after 1 phone call, they will receive a letter in the mail requesting that they complete the survey and will also be given the option to complete the survey by email. At this point, the participant's

clinician will be asked if there have been changes to contact information in a PSS message. The clinician will be asked to provide the patient with the telephone number that they can call in order to complete the survey. After these steps, if the patient has not completed the survey, they will be called again and given the option to complete the survey in person at the clinic, or in person at their home. If the participant is not reached, or for those who agreed to the home visit and it was not successful (for instance, the patient is not home or does not answer the door) a letter will be left at the home or mailed to the address to inform them we have tried to reach them and to remind them about the survey.

There is no "gold standard" measure of medication adherence because more invasive measures of adherence can change actual adherence. We will employ questionnaires, chart reviews and electronic monitoring as recommended and previously used in trials[38]. Standard and validated medication adherence questions will be completed by patients over the telephone, by email, or during their regularly scheduled clinic visits between 9 and 18 months and again between 21 and 30 months to determine the proportion of reported missed doses ("In the last week, how many times did you miss taking your regular medications?").[38] Self-reported non-adherence, taking into consideration the number of expected medication doses, greater than 80 % will be considered adherent (e.g. one or zero missed doses in one week for a medication taken once a day). While prone to memory bias as patients attempt to recall their adherence patterns[3], questionnaires are not as susceptible to social desirability bias as interviews due to anonymity[3, 38] and do not suffer from a drop in completion rates as a study progresses, as diaries do[39]. Chart reviews will be conducted by accessing electronic medical records (EMRs) to determine if long term medications are re-ordered when expected (i.e. for patients who are prescribed a three month supply of medication, evidence of written prescriptions every three months would indicate adherence) in a blinded fashion. Prescriptions written within 18 days of the expected renewal date (20 % of typical renewal period of 90 days) will be classified as adherent. While susceptible to pill dumping, like other objective measures such as pill counts[3], EMR reviews do not depend on recall. Electronic monitoring is considered by many the best method for measuring adherence and is the only method that can practicably provide daily dosage information[40]. However, given the high costs involved only a small subset of patients in the study will be measured in this way[40]. If bottle openings to take medications take place greater than 80% of the times expected, a patient will be classified as adherent. Self-reporting has been shown to overestimate adherence[3] while electronic monitoring is prone to underestimation[40].

This combination of three methods for assessing adherence will be more rigorous than the single methods used in previous trials. [29, 41, 42]

Adherence will be treated as a dichotomous variable. Participants deemed non-adherent by any of the three methods will be classified as non-adherent for the primary analysis. We will also report adherence rates by each of the three measures for the intervention and control groups. Prescriptions for medications intended to be taken on an "as needed" basis (e.g. analgesics, salbutamol) will be excluded from the adherence analysis.

Prescribing appropriateness will be assessed by blinded assessors with access to patient electronic medical records including clinician notes. Two independent adjudicators will verify all prescriptions for appropriateness. Any disputes will be resolved by a third independent adjudicator. Assessors will review all prescriptions written to patients in control and intervention arms using independently developed explicit patient level prescribing appropriateness

indicators.[43] These indicators address the management of common chronic diseases such as heart disease, diabetes and asthma.[43] Prescribing appropriateness is a dichotomous variable and thus each prescription will be counted as appropriate or not appropriate.

The ***primary outcome of appropriate adherence*** will be determined by assessing the percentage of patients who were both adherent to all prescribed medications and received no inappropriate prescriptions.

Secondary outcomes

Adherence and appropriate prescribing will each also be secondary outcomes. For each, we will report the proportion of all prescriptions in each arm that meet the above criteria.

The mean change in HbA1c for patients with diabetes will be a secondary outcome. HbA1c levels will be mandatorily determined between 9 and 12 months and between 21 and 24 months after randomization in patients who had an HbA1c level measured no more than 6 months before randomization and who were prescribed a treatment for diabetes (e.g. metformin, gliclazide, or insulin). HbA1c is easy to assess, is routinely measured and is known to be associated with clinically important outcomes such as myocardial infarction and mortality[44].

The mean change in blood pressure for patients with hypertension or diabetes will be a secondary outcome. This will be assessed mandatorily between 9 and 12 months and between 21 and 24 months after randomization in patients who had a blood pressure reading recorded no more than 6 months before randomization and who are prescribed an antihypertensive treatment. Blood pressure is routinely measured and is known to be associated with clinically important outcomes such as myocardial infarction,[45] stroke[46] and mortality.[47]

The mean change in low density lipoprotein cholesterol (LDL) will be a secondary outcome. LDL will be determined mandatorily between 9 and 12 months and between 21 and 24 months after randomization in patients who had an LDL level measured within 12 months of randomization and who are prescribed a statin. LDL levels are routinely measured and known to be associated with clinically important outcomes such as myocardial infarction, stroke and mortality [48]. Reductions in LDL by statins and other treatments reduce the risk of death based on multiple statin trials and preliminary results from the IMPROVE-IT trial (ezitimibe-simvastatin versus statin) presented in November of 2014 [49].

HbA1c, blood pressure and LDL will be assessed by audits of the EMR.

Patient and community engagement are an important and large part of this study and separate funding has been obtained from St Michael's Hospital and the Toronto Central Local Health Integration Network for engagement around the development of intervention and the selection of outcomes. Briefly, patient, clinician and pharmacist concerns and experiences with the dispensing model will be collected using questionnaires, focus groups and one-on-one interviews. In addition, focus groups and patient panels organized in collaboration before, during and after the study will provide feedback on the intervention.

An analysis of medication costs will be performed as described below.

2.10 Will health service research issues be addressed?

A cost analysis of per capita medication costs between control and intervention arms will be conducted. The analysis cannot be described in detail here due to space restrictions. Briefly, we will determine the mean medication costs per patient and compare this between the intervention and control groups. We will use these data to estimate the cost implications of widespread implementation of this model using Ontario prescribing data (for public assistance recipients) from the Institute of Clinical and Evaluative Sciences and Canadian prescribing data from IMS

Brogan. We will also assess any implications of health outcome changes (including projected impact on clinical outcomes such as mortality) and changes in healthcare resource limitation. The team includes several health economists (see section 3.2).

2.11 What is the proposed sample size and justification?

Based on previous studies [29, 41, 42] we expected that between 40% and 60% of patients in the control group will be appropriately adherent. We expect that at least 90% of patients in the intervention group will be compliant (i.e. accept the free medications offered in the intervention). We believe that a 10% absolute improvement in appropriate adherence (in the presence of non-compliance) is the minimal difference that is clinically important. Assuming a Type I error probability of 5%, a sample size of 392 per group is required to have 80% power to detect a 10% absolute difference in adherence for any control group adherence values between 40% and 60%. No inflation for drop-out is applied since drop-outs will be considered non-adherent. Based on previous trials we expect the drop-out rate to be approximately 5 %.[29, 41, 42]

2.12 What is the planned recruitment rate? How will the recruitment be organized?

We project a recruitment rate of 75 patients per week based on a chart review and on completed RCTs.[29,48,49] Thus recruitment should require less than three months.

Participants will be recruited during routine primary care visits. Based on our chart review, primary care providers see at least 320 unique patients per week (approximately 4 per hour) and approximately 20 % of encounters involve a prescription for a medication. If 20 % of these 64 patients per week who receive a prescription medication are approached by an embedded study research assistant (32 approaches per week), and 20 % are eligible (i.e. report cost-related nonadherence), then 2-4 patients per clinician will be eligible to participate. If 50 % of eligible patients agree to enroll in the study (to have a chance of receiving medications for free), each clinician will recruit approximately 1-2 patients per week.

There are a total of 50 full time equivalent clinicians at the three sites. Thus approximately 75 patients can be recruited each week.

This estimate is conservative as it assumes: that approaches will only take place in encounters involving a prescription medication while care providers may choose to approach patients at other encounters (e.g. a patient on a long term medication who has not presented for a prescription), a moderate approach rate, a low rate of self-reported cost-related non-adherence and a low rate of agreement to participate in a trial of receiving medications for free.

2.13 Are there likely to be any problems with compliance?

The primary outcome is medication adherence by patients. In this trial, “compliance” with the intervention refers to participants deciding to receive the essential medications without charge rather than taking the medications they were previously taking. We anticipate that few patients will choose to enroll in this study but then choose not to take *any* medications provided for free. Some participants may choose to take some medications from the list of essential medications and to also take medications that are not on the list (and this will not pose a problem for the trial). For the sample size calculation, we have assumed a compliance rate of 90 % for the intervention group.

2.14 What is the likely rate of loss to follow up?

We expect a low loss to follow up rate because participants are established patients of family practices. Medication adherence from written prescriptions, prescribing appropriateness and cost analyses can be assessed using information extracted directly from electronic medical records

regardless of patient follow-up. The rate of being lost to follow up ranged from 5 to 16 % in the control group and between 5 and 11 % in the intervention group of previous trials.

Study	Control	Intervention	Reasons
Choudhry 2011	151/3010 (5.0 %)	133/2845 (4.7 %)	Participants lost insurance coverage
Farooq 2011	9/55 (16.4 %)	6/55 (10.9 %)	Drop out, died, discontinued intervention, withdrew consent
Thom 2013	50/1002 (5.0 %)	47/1002 (4.7 %)	Deaths, refused further participation, unable to contact, other

If a participant leaves the primary care practice, we will attempt to obtain the name of the new care provider and obtain the chart for review. If a participant has left the primary care practice and we are unable to contact them, we will censor the data from that participant after they have left the primary care practice.

2.15 How many centers will be involved?

The trial will be conducted in one urban family practice affiliated with St. Michael's Hospital in Toronto and two rural family practices in Ontario: Blind River and Manitoulin Island. All sites meet specifications including the use of the same electronic medical record system.

2.16 What is the proposed type of analyses?

Patient demographics will be summarized descriptively (e.g. means and SD or median IQR for continuous variables and frequency and percentages for categorical). Although randomization guarantees balance in the long-run, there is a chance of imbalances in any sample. The demographics will be reviewed for clinically important imbalances that may be adjusted for in a secondary analysis.

The primary analysis will be performed on an intention to treat basis. All patients can be included since drop-outs or withdrawals will be considered non-adherent. Appropriate adherence will be compared using a chi-square test and the unadjusted treatment effect will be expressed as the absolute risk difference with 95% confidence interval. For the primary analysis a p-value of <0.05 will be sufficient to reject the null hypothesis of no difference. Adjusted analyses will employ logistic regression and the adjusted (conditional) treatment effect will be expressed as an odds ratio with 95% confidence interval.

The first two secondary outcomes are binary at the level of prescription within patient. This will be aggregated to the patient level and logistic regression will be used to compare groups. HbA1C, blood pressure and LDL cholesterol are all continuous and measured at baseline and 9-12 months. An analysis of covariance (ANCOVA) model will be used (via linear regression) to provide the estimates of treatment effect adjusted for baseline values. Since these are all secondary analyses which are supportive or exploratory, no corrections for multiple testing will be applied and only patients with data will be included. However the ITT approach is still followed as patients will remain allocated to their randomized groups irrespective of compliance.

2.17 What is the proposed frequency of analyses?

Analysis will occur at the completion of the study when all data are collected. No interim analyses are planned.

2.18 Are there any planned subgroup analyses?

We will determine if the effects on medication adherence and health outcomes differ by age and sex.

2.19 Has any pilot study been carried out using this design?

The intervention is complex and substantial resources have been dedicated to its development. We have verified the feasibility of chart reviews similar to those that will be used for outcome ascertainment. We believe that a full trial of the intervention is warranted (see *section 1.1*).

3. Trial Management

3.1 What are the arrangements for the day to day management of the trial?

The study will be coordinated from St Michael's Hospital in Toronto. The clinical sites will have site research coordinators who will be responsible for recruitment, training of clinicians and patients, and data collection for the duration of the project. They will liaise with a clinical research coordinator in Toronto who will review their data and provide further instructions as required. The lead clinical research coordinator will also be responsible for liaising with the pharmacist, REB board, principal investigators and safety committees. The research team will receive trial implementation assistance from the Applied Health Research Centre (AHRC) who will advise on trial coordination, site training, site start-up/activation, document management, supply management, database development, data management and statistical analysis. The AHRC has experience managing more than 50 multi-site, national and international studies including 15 CIHR-funded multi-centre trials and has grown to be one of the largest academic research organizations in Canada.

Study data and patient questionnaires will be entered and maintained on a secure password protected database developed using Medidata RAVE (www.mdsol.com) and will be accessible via the internet for data entry purposes. Quality and completeness of data entry will be reviewed as soon as possible after data entry, within 5 business days of data entry for the first 5 participants randomized at each site, and within 15 days of data entry thereafter. Corrections or changes in the data management system will be tracked with the retention of the original data and the corrected data with the date of data entry and submitting personnel.

3.2 What will be the role of each principal applicant and co-applicant proposed?

The co-principal investigator Andreas Laupacis (Canada Research Chair in Health Policy and Citizen Engagement and Executive Director, Li Ka Shing Knowledge Institute) is mentoring the principal applicant Nav Persaud through a CIHR RCT training grant to develop this trial.

AL and NP will together oversee all aspects of the project with input from all co-investigators. Some relevant experiences of the co-investigators are briefly highlighted here. Muhammad Mamdani (Director of the Applied Health Research Centre), Stephen Hwang (Scientist at the Centre for Research on Inner City Health), and Andrew Pinto (Associate Scientist, MSc Health Policy, Planning and Financing) have experience conducting RCTs of complex health interventions. Kevin Thorpe (Assistant Professor) is a biostatistician with experience from dozens of RCTs. Richard Glazier (Senior Scientist and Program Lead at the Institute for Clinical Evaluative Sciences), Steve Morgan (PhD in Economics) and Braden Manns (Svare Professor in Health Economics) have experience relevant to the health economics evaluation. Michael Law (PhD in Health Policy) and Dr. Paul Oh (Scientist at the Toronto Rehabilitation Institute) have expertise in pharmaceutical policy that is relevant both to the study design and the knowledge translation plan. Danielle Martin (Masters of Public Policy) and Andrew Boozary (Masters of Public Policy) have experience communicating scientific findings to policymakers.

3.3 Describe the trial steering committee and any data safety and monitoring committee.

The trial steering committee will include the two study principal investigators (NP and AL), a patient representative, a pharmacist, and a biostatistician.

The primary purpose of the data safety and monitoring committee will be to ensure medication errors are properly addressed. Since we expect medication errors to be rare, each medication error will be reported to the DSMB immediately. The DSMB will make recommendations to the research team about how to mitigate the harm from the medication error and how to prevent future similar errors. The DSMB will have the power to recommend that the trial be stopped if there is an excess of medication errors or if identified errors are not appropriately handled.

The chair of the DSMB will be Dr Dee Mangin who is a family physician and research scientist with experience conducting clinical trials in primary care related to prescribing appropriateness.

References

1. Gagnon, M.-A., *A Roadmap to a Rational Pharmacare Policy in Canada*. 2014, School of Public Policy & Administration, Carleton University.
2. Osterberg, L. and T. Blaschke, *Adherence to medication.* N Engl J Med, 2005. **353**(5): p. 487-97.
3. Lehmann, A., et al., *Assessing medication adherence: options to consider.* Int J Clin Pharm, 2014. **36**(1): p. 55-69.
4. Tamblyn, R., et al., *The incidence and determinants of primary nonadherence with prescribed medication in primary care: a cohort study.* Ann Intern Med, 2014. **160**(7): p. 441-50.
5. Law, M.R., et al., *The effect of cost on adherence to prescription medications in Canada.* Cmaj, 2012. **184**(3): p. 297-302.
6. Choudhry, N.K., et al., *Cost-effectiveness of providing full drug coverage to increase medication adherence in post-myocardial infarction Medicare beneficiaries.* Circulation, 2008. **117**(10): p. 1261-8.
7. *Ontario Public Drug Programs - Formulary - Drugs Funded by Ontario Drug Benefit (ODB) Program*. 2014 [cited 2014 8/25/2014]; Available from: http://www.health.gov.on.ca/en/pro/programs/drugs/odbf_mn.aspx.
8. Howard, M., et al., *Prescribing of potentially inappropriate medications to elderly people.* Fam Pract, 2004. **21**(3): p. 244-7.
9. McGettigan, P. and D. Henry, *Use of non-steroidal anti-inflammatory drugs that elevate cardiovascular risk: an examination of sales and essential medicines lists in low-, middle-, and high-income countries.* PLoS Med, 2013. **10**(2): p. e1001388.
10. Bhala, N., et al., *Vascular and upper gastrointestinal effects of non-steroidal anti-inflammatory drugs: meta-analyses of individual participant data from randomised trials.* Lancet, 2013. **382**(9894): p. 769-79.
11. CIHI, *National Health Expenditure Trends, 1975 to 2014*. 2014, CIHI.
12. Gustafsson, L.L., et al., *The 'wise list'- a comprehensive concept to select, communicate and achieve adherence to recommendations of essential drugs in ambulatory care in Stockholm.* Basic & Clinical Pharmacology & Toxicology, 2011. **108**(4): p. 224-33.
13. OECD. *Pharmaceutical expenditure per capita*. 2014 2/20/2015]; Available from: http://www.oecd-ilibrary.org/social-issues-migration-health/pharmaceutical-expenditure-per-capita_pharmexpcap-table-en.
14. Morgan, S.G., J.R. Daw, and M.R. Law, *Rethinking pharmacare in Canada*. 2013: CD Howe Institute.
15. Gagnon, M.D. and G. Hebert, *The Economic Case for Universal Pharmacare: Costs and Benefits of Publicly Funded Drug Coverage for all Canadians*. 2010.
16. Wu, C., C.M. Bell, and W.P. Wodchis, *Incidence and economic burden of adverse drug reactions among elderly patients in Ontario emergency departments: a retrospective study.* Drug Saf, 2012. **35**(9): p. 769-81.
17. Zhang, L., et al., *Mail-order pharmacy use and medication adherence among Medicare Part D beneficiaries with diabetes.* Journal of Medical Economics, 2011. **14**(5): p. 562-7.
18. Adams, A.S., et al., *Health system factors and antihypertensive adherence in a racially and ethnically diverse cohort of new users.* JAMA Internal Medicine, 2013. **173**(1): p. 54-61.
19. Duru, O.K., et al., *Mail-order pharmacy use and adherence to diabetes-related medications.* American Journal of Managed Care, 2010. **16**(1): p. 33-40.

20. Johnsrud, M., K.A. Lawson, and M.D. Shepherd, *Comparison of mail-order with community pharmacy in plan sponsor cost and member cost in two large pharmacy benefit plans.* Journal of Managed Care Pharmacy, 2007. **13**(2): p. 122-34.
21. Visaria, J., et al., *Comparing the cost of community pharmacy and mail-order pharmacy in a US retirement system.* International Journal of Health Planning & Management, 2012. **27**(1): p. e41-50.
22. Schmittdiel, J.A., et al., *Safety and effectiveness of mail order pharmacy use in diabetes.* American Journal of Managed Care, 2013. **19**(11): p. 882-7.
23. Schmittdiel, J.A., et al., *The comparative effectiveness of mail order pharmacy use vs. local pharmacy use on LDL-C control in new statin users.* Journal of General Internal Medicine, 2011. **26**(12): p. 1396-402.
24. Beardon, P.H., et al., *Introducing a drug formulary to general practice--effects on practice prescribing costs.* J R Coll Gen Pract, 1987. **37**(300): p. 305-7.
25. Romanow, R.J., *Building on Values: The Future of Health Care in Canada - Final Report*. 2002, Government of Canada.
26. Kirby, M.J. and M. LeBreton, *The Health of Canadians - The Federal Role - Final Report - Volume Six: Recommendations for Reform*. 2002, The Standing Senate Committee on Social Affairs, Science and Technology.
27. Hoskins, E., *Eric Hoskins: The time for national pharmacare has come*, in *Toronto Star*. 2014: Toronto.
28. Nieuwlaat, R., et al., *Interventions for enhancing medication adherence.* Cochrane Database Syst Rev, 2014. **11**: p. Cd000011.
29. Choudhry, N.K., et al., *Full coverage for preventive medications after myocardial infarction.* New England Journal of Medicine, 2011. **365**(22): p. 2088-97.
30. Hill-Smith, I., *Sharing resources to create a district drug formulary: a countywide controlled trial.* British Journal of General Practice, 1996. **46**(406): p. 271-75.
31. Avery, A.J., et al., *Do prescribing formularies help GPs prescribe from a narrower range of drugs? A controlled trial of the introduction of prescribing formularies for NSAIDs.* British Journal of General Practice, 1997. **47**(425): p. 810-4.
32. WHO. *DDD - Definitions and general considerations*. 2009 2009/12/17 2015/2/13]; Available from: http://www.whocc.no/ddd/definition_and_general_considera/.
33. Kesselheim, A.S., et al., *Clinical equivalence of generic and brand-name drugs used in cardiovascular disease: a systematic review and meta-analysis.* Jama, 2008. **300**(21): p. 2514-26.
34. Kesselheim, A.S., et al., *Seizure outcomes following the use of generic versus brand-name antiepileptic drugs: a systematic review and meta-analysis.* Drugs, 2010. **70**(5): p. 605-21.
35. Guberman, A. and C. Corman, *Generic substitution for brand name antiepileptic drugs: a survey.* Can J Neurol Sci, 2000. **27**(1): p. 37-43.
36. Faasse, K., et al., *The effect of an apparent change to a branded or generic medication on drug effectiveness and side effects.* Psychosomatic Medicine, 2013. **75**(1): p. 90-6.
37. Canada, H. *Canadian Community Health Survey*. 2012 2012/04/23 2015/2/13]; Available from: http://www.hc-sc.gc.ca/fn-an/surveill/nutrition/commun/index-eng.php.
38. Garber, M.C., et al., *The concordance of self-report with other measures of medication adherence: a summary of the literature.* Med Care, 2004. **42**(7): p. 649-52.
39. Butz, A.M., et al., *Monitoring nebulizer use in children: comparison of electronic and asthma diary data.* Ann Allergy Asthma Immunol, 2005. **94**(3): p. 360-5.
40. Cook, P., et al., *Practical and analytic issues in the electronic assessment of adherence.* West J Nurs Res, 2012. **34**(5): p. 598-620.

41. Farooq, S., et al., *Schizophrenia medication adherence in a resource-poor setting: randomised controlled trial of supervised treatment in out-patients for schizophrenia (STOPS).* Br J Psychiatry, 2011. **199**(6): p. 467-72.
42. Thom, S., et al., *Effects of a fixed-dose combination strategy on adherence and risk factors in patients with or at high risk of CVD: the UMPIRE randomized clinical trial.* Jama, 2013. **310**(9): p. 918-29.
43. Fernandez Urrusuno, R., et al., *Compliance with quality prescribing indicators in terms of their relationship to financial incentives.* European Journal of Clinical Pharmacology, 2013. **69**(10): p. 1845-53.
44. Macisaac, R.J. and G. Jerums, *Intensive glucose control and cardiovascular outcomes in type 2 diabetes.* Heart, Lung & Circulation, 2011. **20**(10): p. 647-54.
45. Papademetriou, V., *From Hypertension to Heart Failure.* The Journal of Clinical Hypertension, 2004. **6**: p. 14-17.
46. Nagai, M. and K. Kario, *Visit-to-visit blood pressure variability, silent cerebral injury, and risk of stroke.* Am J Hypertens, 2013. **26**(12): p. 1369-76.
47. Briasoulis, A., et al., *Effects of antihypertensive treatment in patients over 65 years of age: a meta-analysis of randomised controlled studies.* Heart, 2014. **100**(4): p. 317-23.
48. Stone, N.J., et al., *Treatment of blood cholesterol to reduce atherosclerotic cardiovascular disease risk in adults: synopsis of the 2013 American College of Cardiology/American Heart Association cholesterol guideline.* Ann Intern Med, 2014. **160**(5): p. 339-43.
49. Blazing, M.A., et al., *Evaluating cardiovascular event reduction with ezetimibe as an adjunct to simvastatin in 18,144 patients after acute coronary syndromes: final baseline characteristics of the IMPROVE-IT study population.* Am Heart J, 2014. **168**(2): p. 205-12.e1.

Summary of changes to CLEAN Meds protocol

The duration of the trial was extended from 12 months to 24 months when additional funding was secured. The primary analysis was done at 12 months.

For the analysis of the primary outcome, we originally planned to use electronic pill bottle cap devices in one-seventh of participants to confirm adherence measurements but due to a large amount of missing data in both arms, we removed this from the definition of the primary outcome. We also report the results when the available electronic pill bottle cap device data was used.

We allowed the study pharmacist to deliver medicines to a community pharmacy (instead of the patient's home) if this was requested.
