# Supplementary material for: Effect on Treatment Adherence of Distributing Essential Medicines at No Charge: The CLEAN Meds Randomized Clinical Trial
Source: JAMA Intern Med. 2019 Oct 7;180(1):27–34. doi: 10.1001/jamainternmed.2019.4472 (PMC6784757; doi:10.1001/jamainternmed.2019.4472)
Supplement: Supplement 2. — eAppendix 1. Essential Medicines Provided to Patients With Free Distribution eAppendix 2. Criteria for Potentially Inappropriate Prescriptions eTable 1. Alternative Definitions of Primary Outcome Results Table eTable 2. Patient Oriented Outcome Results Table [file jamainternmed-180-27-s002.pdf]

Supplementary Online Content

Persaud N, Bedard M, Boozary AS, et al; Carefully Selected and Easily Accessible at No Charge Medicines (CLEAN Meds) Study Team. Effect on treatment adherence of distributing essential medicines at no charge: the CLEAN Meds randomized clinical trial. *JAMA Intern Med.* Published online October 7, 2019.
doi:10.1001/jamainternmed.2019.4472

eAppendix 1. Essential Medicines Provided to Patients With Free Distribution
eAppendix 2. Criteria for Potentially Inappropriate Prescriptions
eTable 1. Alternative Definitions of Primary Outcome Results Table
eTable 2. Patient Oriented Outcome Results Table

This supplementary material has been provided by the authors to give readers additional information about their work.

© 2019 Persaud N et al. *JAMA Internal Medicine*.

eAppendix 1. Essential Medicines Provided to Patients With Free Distribution

- acetaminophen
- acetylsalicylic acid
- adalimumab
- alendronate
- allopurinol
- amiodarone
- amlodipine
- amoxicillin
- amoxicillin/clavulanic acid
- atomoxetine
- atorvastatin
- azathioprine
- azithromycin
- baclofen
- beclomethasone
- benzoyl peroxide
- benztropine
- betamethasone
- bevacizumab
- bisoprolol
- budesonide
- candesartan
- carbamazepine
- cephalexin
- cetirizine
- chlorthalidone
- ciprofloxacin
- clarithromycin
- clindamycin
- clopidogrel
- clotrimazole
- cloxacillin
- clozapine
- conjugated estrogens
- copper (intrauterine device)

© 2019 Persaud N et al. *JAMA Internal Medicine*.

- dabigatran
- dexamethasone
- diltiazem
- dolutegravir
- domperidone
- donepezil
- doxycycline
- efavirenz
- eletriptan
- emtricitabine
- epinephrine
- estradiol
- ethinyl estradiol/levonorgestrel
- ferrous fumarate
- finasteride
- fluconazole
- fluoxetine
- fluticasone
- folic acid
- furosemide
- fusidic acid
- gabapentin
- gliclazide
- haloperidol
- hydrocortisone
- hydroxychloroquine
- ibuprofen
- insulin, long acting
- insulin, short acting
- ipratropium
- labetalol
- lamivudine
- latanoprost
- levodopa/carbidopa
- levofloxacin
- levonorgestrel
- levothyroxine
- lithium

© 2019 Persaud N et al. *JAMA Internal Medicine*.

- loperamide
- medroxyprogesterone
- metformin
- methimazole
- methotrexate
- methylprednisolone
- metoclopramide
- metronidazole
- mupirocin
- naltrexone
- naproxen
- nicotine replacement therapy
- nitrofurantoin
- nitroglycerin
- nortriptyline
- nystatin
- olopatadine
- pantoprazole
- permethrin
- phenytoin
- pilocarpine
- polyethylene glycol 3350
- polymyxin B
- potassium
- pravastatin
- prednisone
- propylthiouracil
- ramipril
- ranitidine
- risperidone
- rivaroxaban
- salbutamol
- salicylic acid
- salmeterol
- senna
- sertraline
- spironolactone
- sulfamethoxazole/trimethoprim

© 2019 Persaud N et al. *JAMA Internal Medicine*.

- sulfasalazine
- tadalafil
- tamsulosin
- tenofovir disoproxil fumarate
- thiamine
- tiotropium
- tranexamic acid
- tretinoin
- trimethoprim
- urea
- vaginal ring eluting etonogestrel and ethinyl estradiol
- valacyclovir
- valproic acid
- varenicline
- vitamin B12
- vitamin D
- warfarin

eReference

Taglione MS, Ahmad H, Slater M, Aliarzadeh B, Glazier RH, Laupacis A, Persaud N. Development of a preliminary essential medicines list for Canada. CMAJ Open. 2017 Feb 16;5(1):E137-E143.

© 2019 Persaud N et al. *JAMA Internal Medicine*.

eAppendix 2. Criteria for Potentially Inappropriate Prescriptions

The following criteria was applied to medicine lists for each patient.

- beta-blocker and cardioselective calcium channel blocker
- warfarin and non-steroidal anti-inflammatory
- direct-acting anticoagulant and non-steroidal anti-inflammatory
- warfarin and acetylsalicylic acid
- nitrate and phosphodiesterase inhibitor
- acetylsalicylic acid at doses greater than 160 mg per day
- glyburide at any dose (alternatives exist)
- duplicate class prescription (e.g. two thiazide diuretics simultaneously or two calcium channel blockers simultaneously)

eReference

O'Mahony D, O'Sullivan D, Byrne S, O'Connor MN, Ryan C, Gallagher P. STOPP/START criteria for potentially inappropriate prescribing in older people: version 2. *Age Ageing*. 2014;44(2):213-8.

© 2019 Persaud N et al. *JAMA Internal Medicine*.

eTable 1. Alternative Definitions of Primary Outcome Results Table

	Free distribution (N=395)	Usual access (N=391)	Difference	*P* value
Participants adherent to all appropriately prescribed medicines (exclude any inappropriate prescriptions)	152/395 (38.5 %)	106/391 (27.1 %)	11.4 %; 95 % CI 4.6 % to 18.1 %	0.00091
Participants appropriately adherent to all medicines, excluding those with no regular medicines (those taking only as needed medicines)	151/349 (43.3 %)	104/322 (32.3 %)	11.0 %; 95 % CI 3.4 % to 18.6 %	0.0044
Participants appropriately adherent to all medicines, excluding those who withdrew	151/387 (39.0 %)	104/376 (27.7 %)	11.4 %; 95 % CI 4.5 % to 18.3 %	0.0012
Participants appropriately adherent to all medicines, using only patient report data	183/216 (84.7 %)	135/179 (75.4 %)	9.3 %; 95 % CI 8.7 % to 17.7 %	0.028
Participants appropriately adherent to all medicines, using only chart review data	154/315 (48.9 %)	109/281 (38.8 %)	10.1 %; 95 % CI 1.8 % to 18.4 %	0.016
Participants appropriately adherent to all medicines, using electronic bottle cap data in addition to patient report and chart review data	150/395 (38.0)	104/391 (26.6)	11.4 %; 95 % CI 4.6 % to 18.1 %	0.00086

© 2019 Persaud N et al. *JAMA Internal Medicine*.

eTable 2. Patient Oriented Outcome Results Table

	Free distribution, N (%) (N=395)	Usual access, N (%) (N=391)	difference	*P* value
Did you feel your care was better than before the study started?	123/266 (46.2)	47/251 (18.7)	27.5 %; 95 % CI 19.4 % to 35.6 %	<0.0001
Did you feel your questions or concerns were addressed by health care provider?	252/267 (94.4)	220/248 (88.7)	5.7 %; 95 % CI 0.5 % to 10.9 %	0.030
Was your interaction with specialists different from before the study started?	51/267 (19.1)	28/252 (11.1)	8.0 %; 95 % CI 1.5 % to 14.5 %	0.016
Did the information about medications given by the pharmacist and the doctor match?	256/268 (95.5)	218/240 (90.8)	4.7 %; 95 % CI -0.12 % to 9.5 %	0.053
Was it easy to obtain information about the medications?	265/269 (98.5)	235/248 (94.8)	3.8 %; 95 % CI 0.24 % to 7.3 %	0.032
Did the information change the way you took medications?	83/267 (31.1)	63/245 (25.7)	5.4 %; 95 % CI -2.8 % to 13.6 %	0.212
Did you have unanswered questions about your medications?	12/267 (4.5)	20/248 (8.1)	-3.4 %; 95 % CI -8.2 % to 1.0 %	0.135
Was the medication in good condition when you received it?	265/268 (98.9)	231/242 (95.5)	3.4 %; 95 % CI 0.12 % to 6.7 %	0.036
Did you receive new medications quickly?	239/260 (91.9)	207/239 (86.6)	5.3 %; 95 % CI -0.53 % to 11.2 %	0.075
Did you receive the medication before the previous prescription ran out?	217/261 (83.1)	157/237 (66.2)	16.9 %; 95 % CI 9.0 % to 24.8 %	<0.0001

© 2019 Persaud N et al. *JAMA Internal Medicine*.

Was it easier to make ends meet at the end of the month?	230/266 (86.5)	79/238 (33.2)	53.3 %; 95 % CI 45.6 % to 60.9 %	<0.0001
Do you feel your health improved?	207/266 (77.8)	122/239 (51.0)	26.8 %; 95 % CI 18.3 % to 35.2 %	<0.0001
Did you have any medication side effects?	72/267 (27.0)	86/244 (35.2)	-8.3 %; 95 % CI -16.7 % to 0.13 %	0.054
Did you have fewer healthcare visits?	128/264 (48.5)	81/242 (33.5)	15.0 %; 95 % CI 6.2 % to 23.9 %	0.00085

© 2019 Persaud N et al. *JAMA Internal Medicine*.
