# Supplementary material for: Effect on Treatment Adherence of Distributing Essential Medicines at No Charge: The CLEAN Meds Randomized Clinical Trial
Source: JAMA Intern Med. 2019 Oct 7;180(1):27–34. doi: 10.1001/jamainternmed.2019.4472 (PMC6784757; doi:10.1001/jamainternmed.2019.4472)
Supplement: Supplement 3. — Data Sharing Statement [file jamainternmed-180-27-s003.pdf]

Data Sharing Statement

Persaud. Effect on Treatment Adherence of Distributing Essential Medicines at No Charge. *JAMA Intern Med*. Published October 07, 2019. 10.1001/jamainternmed.2019.4472

Data
Data available: Yes
Data types: Deidentified participant data, Data dictionary
How to access data: Nav.persaud@utoronto.ca
When available: beginning date: 12-31-2019

Supporting Documents
Document types: None

Additional Information
Who can access the data: No restrictions.
Types of analyses: For any non-commercial purpose.
Mechanisms of data availability: Signed data access agreement.
